# Integrated pathway analysis identifies prognostically relevant subtypes of glioblastoma characterized by abnormalities in multi‐omics

**DOI:** 10.1002/ctm2.70517

**Published:** 2025-11-25

**Authors:** Pei Zhang, Dan Liu, Tonghui Yu, Yanlin Zhang, Lu Zhong, Xiao Ouyang, Qin Xia, Lei Dong

**Affiliations:** ^1^ Advanced Technology Research Institute, State Key Laboratory of Molecular Medicine and Biological Diagnosis and Treatment (Ministry of Industry and Information Technology), School of Life Science Beijing Institute of Technology Beijing China; ^2^ International Center for Interdisciplinary Statistics, School of Mathematics Harbin Institute of Technology Harbin China

**Keywords:** drug sensitivity, machine learning, multi‐omics, pathway‐based subtypes

## Abstract

**Background:**

Gene expression‐based molecular subtypes in glioblastoma from The Cancer Genome Atlas Network (TCGA‐GBM) unraveled the pathological origins by identifying tumour cell driver genes. However, the causal inference between molecular subtype origins and their therapeutic efficacy remains obscure.

**Methods:**

We integrated TCGA‐GBM multi‐omics (DNA, mRNA, and protein profiles) using correlation analysis to identify *cis‐*regulation. We analyzed the exposure‐mediated base substitution‐level mutations and their potential triggers. Importantly, we performed Consensus Clustering based on the MSigDB database with Silhouette‐correction to identify prognostically relevant pathway‐based MSig subtypes. The tumour driver mutations (co‐occurrence mutation pattern), aberrant pathways (tumour hallmarks), immune microenvironment (*xCell*), and pseudo‐time analysis (*dyno*) were used to characterize the MSig subtype landscape. Furthermore, we evaluated potential drug sensitivities across MSig subtypes using the Genomics of Drug Sensitivity in Cancer database.

**Results:**

We classified five MSig subtypes, characterized by neural‐like, tumour‐driving, low tumour evolution, immune‐inflamed, and classical tumour features. We observed several key features in ‘tumour‐driving’ GBM patients: (1) mutual exclusivity between prognostic factors TP53 and EGFR; and (2) IDH1 mutations co‐occurring with TP53, which account for the protective role of IDH1 in TP53 mutant patients. The immune‐inflamed GBM, characterized as a ‘hot’ tumour, exhibited upregulation of immune‐related pathways, including PD‐1 and IFN‐γ signalling responses. DNA methylation landscape revealed 14 MGMT CpG‐rich regions regulating expression. Evolutionary trajectories revealed progression from a primary tumour state (close to normal tissue) to two distinct endpoints (tumour‐driving and immune‐inflamed subtypes).

**Conclusions:**

Our findings reveal interactions between tumour cells and their surrounding immune environment, classifying GBM into two newly identified subtypes: (1) the tumour‐driving subtype is characterized by multiple oncogenic mutations, while (2) the immune‐blockade subtype is marked by a high presence of immune cells. We highlight the importance of integrating multi‐type data (somatic mutations, DNA methylation, and RNA transcripts, etc.) to decipher GBM biology and potential therapeutic implications.

**Highlights:**

We report the interaction between tumor cells and environmental immune cells, classifying GBM into two main subtypes: 1) The tumor‐driving subtype is characterized by multiple oncogenic mutations, while 2) the immune‐blockage subtype is marked by a high presence of immune cells. We used integrated multidimensional analyses of somatic mutations, DNA methylation, and RNA transcripts to gain a deeper understanding of GBM biology and potential therapeutic implications.

## Graphical abstract

We report the interaction between tumour cells and environmental immune cells, classifying GBM into two main subtypes: (1) The tumour‐driving subtype is characterized by multiple oncogenic mutations, while (2) the immune‐blockage subtype is marked by a high presence of immune cells. We used integrated multi‐dimensional analyses of somatic mutations, DNA methylation, and RNA transcripts to gain a deeper understanding of GBM biology and potential therapeutic implications.

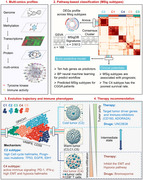

## 1 INTRODUCTION

Glioblastoma (GBM) is the most lethal primary brain tumour in adults, with a median survival of less than 15 months, highlighting the urgent need for more effective therapeutic strategies.^1^, ^2^, ^3^, ^4^, ^5^ Uncovering tumour heterogeneity and identification of prognostically relevant molecular classification is the key to improving therapeutic outcomes for personalized treatments.^4^, ^6^ Previous studies on multi‐omics from The Cancer Genome Atlas Research Network (TCGA) and the Chinese Glioma Genome Atlas (CGGA) databases have identified several key aberrant signalling pathways, such as mutations in IDH, PTEN, TP53, TERT, ATRX, and EGFR,^6^, ^7^, ^8^ that are strongly associated with clinical outcomes. Additionally, the ISN Haarlem guidelines recommended a hierarchical diagnostic approach that integrates histological classification, WHO grade, and molecular classification for a comprehensive assessment and treatment strategy.^9^ These findings underscore the pivotal role of molecular classification in elucidating GBM heterogeneity.

Further evidence shows that single‐omics‐based classifications are insufficient for understanding how tumour heterogeneity interconnects with patient prognosis. For example, the 2016 WHO brain tumour classification delineated GBM into IDH‐wild‐type (IDH‐WT; ∼90%) or IDH‐mutant (∼10%), with the latter associated with better overall survival and increased sensitivity to temozolomide.^1^, ^10^, ^11^, ^12^, ^13^ The status of MGMT promoter methylation also accounts for sensitivity to temozolomide treatment primarily in non‐recurrent classical GBM subtypes.^14^ Additionally, Verhaak et al.^5^ identified four gene expression‐based subtypes (GeneExp‐Subtypes) by integrating genomic and transcriptomic data to demonstrate cell origin: (1) proneural (characterized by PDGFRA and IDH1 mutations, common in younger patients and secondary GBM); (2) neural (close to normal brain tissue with few mutations); (3) classical (marked by EGFR amplifications/mutations and PTEN deletions); and (4) mesenchymal (defined by NF1 abnormalities, altered neurofibromin expression, and mesenchymal/astrocytic differentiation gene expression). However, the overlap of certain characteristics within GeneExp‐Subtypes usually leads to weak correlation with patient prognosis. For instance, the same patient with different tumour regions may exhibit the Neural subtype, combined with the other three subtypes.^15^ Wang et al.^16^ argue that the ambiguity in defining tumour origins, such as neural subtype, originates from non‐tumour cells in the tumour microenvironment. Neftel et al.^17^ conducted a comprehensive study on glioblastoma by integrating single‐cell RNA sequencing of 401 specimens from TCGA and single‐cell lineage tracing. They developed a unified model to capture cellular states and genetic diversity at the glioblastoma cell level. However, we generally believe that the cell state (NPC‐like, OPC‐like, AC‐like, and MES‐like) classification may not fully reflect patient characteristics and drug responses. This is due to the following reasons: (1) tumour heterogeneity in GBM could mask rare but clinically relevant cellular states; and (2) tumour microenvironment (TME) also accounts for cell state plasticity or therapeutic responses. Thus, we focus on classifying GBMs at the patient level based on pathway‐associated hallmarks while considering both malignant cells and their microenvironmental ecosystems.

Machine‐learning is the powerful tool to uncover the tumour heterogeneity, which may have several challenges in analyzing multi‐omics datasets, such as (1) standardization for technical batch effects and biases;^18^, ^19^ (2) deployment of an independent test to improve benchmark model performance during data preprocessing;^20^, ^21^, ^22^ (3) dimension reduction for multi‐omics data (DNA, RNA, methylation, proteins) to improve inference learning.^20^, ^23^ Recently, the Molecular Signatures Database (MSig DB) has standardized gene sets, including biological and/or oncogenic pathways and disease status, for analyzing tumour heterogeneity.^24^ For instance, Thorsson et al.^25^ utilized immune‐related molecular signatures to classify tumour immune subtypes. Similarly, Lin et al.^26^ identified molecular subtypes and classified prognosis using a metabolism‐related gene expression signature obtained from MSig DB. We generally believe molecular pathways are the classification methodologies for dimension reduction. Multi‐molecular integrated pathway‐based classification offers a comprehensive insight compared with single gene‐based classifiers in identifying GBM heterogeneity.^27^, ^28^ Pathway‐based approach also enables the identification of the biomarker and clinical applications for specific subtypes. The abnormal activation of signalling pathways involving EGFR, PTEN, and TP53 is critical for GBM progression.^5^, ^6^, ^29^, ^30^, ^31^, ^32^, ^33^ Garofano et al.^28^ reported that pathway‐based classification of GBM reveals proliferative/ progenitor, neuronal, mitochondrial, and glycolytic/ plurimetabolic subtypes are sensitive to different pathway inhibitors. Li et al.^34^ identified three gastric cancer subtypes based on pathway clustering, offering new insights into tumour biology and clinical implications. Recently, Wang et al.^35^ extended the integration of MS‐based proteomics to identify GBM subtypes that differ in infiltrating macrophages and specific immune cell type distribution. Kim et al.^36^ revealed neuronal transitions associated with WNT/PCP pathway activation and BRAF kinase activity by proteogenomic characterization of GBM, providing insights into the biological mechanisms of GBM evolution and treatment resistance.

In this study, we explored the impact of pathway‐based classification for clinical implications. We integrated multi‐omics profiles of GBM to identify global tumour abnormalities, and analyzed exposure‐mediated mutations at the base substitution level to explore triggering factors. We identified five distinct MSig subtypes through unsupervised consensus clustering based on integrated molecular pathways. We delineated the landscape of tumour driver mutations, aberrant pathways, the immune microenvironment, and evolutionary trajectories across MSig subtypes. Furthermore, we developed treatment recommendations for MSig subtypes using drug sensitivity data from GBM cells in the Genomics of Drug Sensitivity in Cancer (GDSC) database. We conducted a comprehensive framework to characterize the signature‐based classification for precision therapeutic strategies in GBM. Here, we propose a pathway‐integrated GBM classification and analyze MSig subtype characteristics based on multi‐omics, connecting the subtypes to druggable pathway modules and immune contexts.

## 2 METHODS

### 2.1 Data source

(1) The TCGA‐GBM RNA‐seq (TCGA‐GBM.htseq_fpkm.tsv; *n =* 166), methylation (TCGA‐GBM.methylation450.tsv; *n =* 143), GISTIC2 copy number by genes (*n =* 542), protein expression RPPA (*n =* 215), and phenotype data (TCGA‐GBM.GDC_phenotype.tsv) were downloaded from https://xenabrowser.net/datapages/. The mutation annotation format (MAF) file (*n =* 400) for GBM was queried using TCGAmutations in R. We integrated the omics data types by patient ID.

(2) The CGGA‐GBM RNA‐seq and phenotype data were downloaded from http://www.cgga.org.cn/download.jsp, dataset ID: mRNAseq_325. Only patients with GBM (*n =* 85) were extracted.

(3) Cell lines: We downloaded the cell dataset (RNAseq_GBM_cell_use.csv) from https://cellmodelpassports.sanger.ac.uk/. The RNA‐seq FPKM expression data for GBM cell lines (*n =* 30) included the following lines: 42‐MG‐BA, A172, AM‐38, Becker, CAS‐1, CCF‐STTG1, D‐247MG, D‐263MG, D‐392MG, D‐423MG, D‐542MG, D‐566MG, DBTRG‐05MG, GB‐1, GMS‐10, KS‐1, LN‐229, LN‐405, LNZTA3WT4, M059J, SF126, SF268, SF295, SK‐MG‐1, SNB75, SW1088, T98G, U251, YH‐13, and YKG‐1.

### 2.2 Quality control and preprocessing

Covariates with single‐level or zero variance were excluded from the Cox models; specifically, the predictor ‘IDH1_mut’ was dropped in the IDH‐wildtype satisfied analysis due to a lack of variation. A total of nine samples with missing data were excluded from the analytical cohort.

### 2.3 MSig DB: Molecular signatures database and gene set variation analysis

The Molecular Signatures Database (MSig DB) serves as a comprehensive repository for gene expression signatures,^24^ including hallmark, positional, curated, regulatory target, computational, ontology, oncogenic, immunologic, and cell type signatures. We employ the GSVA package in R to calculate patient enrichment scores of molecular signatures based on the gene sets available in MSig DB.^37^ This approach assigns predefined gene ranks to assess pertinent pathways and molecular mechanisms. The analysis spans a range of gene sets, from a minimum of 5 to a maximum of 5000 genes, culminating in a comprehensive enrichment score matrix (*n =* 31613), referred to as the MSig‐matrix. We conducted differential analysis using limma in R to compare the enrichment scores of molecular signatures between GBM patients (*n =* 166) and normal tissue (*n =* 5) in the MSig‐matrix (*p* < .01, fold change > |2|).

### 2.4 Redundancy filtering of gene signatures

To minimize collinearity among gene sets, we computed pairwise Jaccard similarity on set membership and applied a greedy de‐duplication at Jaccard ≥.60: when two signatures exceeded the threshold, one representative was retained (favouring canonical sources and greater unique content), and the other was removed. This procedure reduced the working library to 18 742 non‐redundant signatures. All downstream clustering and stability analyses (including Hallmark‐only and KEGG/Reactome‐only runs) used this filtered library; full details and tables are provided in the Supporting Information.

### 2.5 CNA‐driven cis regulation

Cis‐regulation was analyzed by determining positive correlations among copy number alterations (CNA), mRNA, and proteins using Pearson correlation in R. Positive correlations were identified for genes present in CNA‐mRNA (9113 genes) and CNA–mRNA–proteins (24 genes). In addition, *p*‐values were calculated to assess the statistical significance of these correlation values (*p* < .05).

### 2.6 Mutation gene and mutational signature

We summarize, analyze, and visualize the MAF data of TCGA‐GBM (*n =* 400). Mutation frequency, mutation type, co‐occurring/mutually exclusive mutations, and tumour mutational burden (TMB) are presented using *maftools*.^38^ Single Base Substitution Signatures (SBS), also known as single nucleotide variants (SNVs), are defined as the replacement of a specific nucleotide base. Considering the pyrimidines in Watson–Crick base pairs, there are only six distinct possible substitutions: C > A, C > G, C > T, T > A, T > C, and T > G. SBS across GBM patients and their trinucleotide sequence contexts were decomposed into three distinct mutation signatures using *maftools* ^38^ in R and MutationalPatterns.^38^ COSMIC Mutational Signatures (version 3.3) showed significant correlation with our newly identified set of SBS signatures, as confirmed by our analyses, achieving a median cosine similarity of.8. The correlation between the percentage of GBM‐SBSs and mRNA levels was determined using Pearson's correlation, identifies genes with strong positive correlations to GBM‐SBS contributions (*p* < .05, *r* > .2). These include SBSA (*n =* 105), SBSB (*n =* 155), SBSC (*n =* 3), SBSD (*n =* 18), SBSE (*n =* 524), and SBSF (*n =* 336) (Table S8).

Bootstrapping (fit_to_signatures_bootstrapped) of MutationalPatterns provides greater confidence by introducing slight modifications to the mutational profile of a sample.^39^ For each iteration, a signature refit is performed, resulting in an estimate of refitting stability. By utilizing bootstrapping (repeated 100 times), we refined 96 types of trinucleotide substitutions and analyzed their relative contributions to previously reported single‐base substitutions (SBSs) from COSMIC v3.2^40^, ^41^ across the five MSig subtypes and patients with distinct mutations in the C2 subtype. The COSMIC signatures were downloaded from: https://cancer.sanger.ac.uk/cosmic/signatures.

### 2.7 Consensus cluster

Clustering is performed on GSVA‐derived pathway activity from RNA‐seq (MSig‐matrix). Multi‐omics integration is applied downstream to mechanistically annotate and validate subtypes (CNV–mRNA–protein cis‐regulation; methylation–expression; COSMIC SBS; RPPA/CPTAC).

The ConsensusClusterPlus package^42^ is utilized for agglomerative PAM clustering based on the MSig matrix, employing a Minkowski distance metric. The process involves resampling 80% of the samples across ten iterations to determine the optimal clustering solution. This determination is made by analyzing an empirical cumulative distribution function plot. To assess and refine the quality of the clustering, the Silhouette coefficient, which ranges from −1 to 1, is employed. Finally, we cluster GBM patients into five MSig subtypes: C1, C2, C3, C4, and C5.

### 2.8 Weighted Gene Co‐expression Network Analysis

Weighted Gene Co‐expression Network Analysis (WGCNA)^43^ is applied to develop a scale‐free co‐expression network utilizing TCGA‐GBM gene expression data. This involves pairwise correlation and average linkage methods for all gene pairs. After selecting an appropriate power, the adjacency matrix is converted into a topological overlap matrix (TOM). The final model comprises ten co‐expression modules, with the grey module denoting genes not assignable to any specific module. These hub genes, characterized by their high connectivity, are crucial in regulating their respective co‐expression networks. Significant correlations were observed between specific MSig subtypes (C1, C2, C3, C4, and C5) and four distinct co‐expression modules (turquoise, yellow, blue, and magenta) (Figure 3F,G). The top‐ten hub genes for each MSig subtype were identified based on gene significance (|GS| > .3) and module membership (|MM| > .8).

### 2.9 Machine learning model

We employed a series of stringent validation methods to ensure the reliability and generalizability of our model: (1) The CGGA cohort was used as an independent hold‐out set to assess the model's performance on external data; (2) five‐fold cross‐validation was implemented on the TCGA dataset to evaluate the model's stability; (3) molecular validation was conducted in GBM cell lines to confirm the accuracy of the subtype assignments. The hub genes were carefully selected based on their differential expression across subtypes, prognostic value in GBM patients, network centrality within the gene co‐expression network, and biological relevance to the characteristic features of each subtype.

### 2.10 Trajectories inference

Utilizing the R *Dyno* package for analysis and visualization of cell differentiation trajectories^44^ (FPKM: Fragments Per Kilobase Million), the procedure encompasses the following steps: (1) Data preparation: Input of gene expression FPKM data from 166 TCGA‐GBM patients. (2) Data preprocessing: Includes quality control and preprocessing of raw data, such as noise removal, normalization, and gene filtering. These steps ensure data accuracy and reliability. (3) Cell state inference: Each cell state is inferred based on gene expression markers specific to cell types and states. (4) Construction of differentiation trajectories: cell differentiation trajectories are constructed using pseudotime algorithms and network graph algorithms, incorporating the inferred cell states as input. (5) Visualization and analysis: The cell differentiation trajectories are visualized graphically. Branch node genes (top 20 genes associated with each trajectory) involved in the transition process are identified to gain deeper insights into the mechanisms of cell differentiation.

### 2.11 Tumour microenvironment and immune‐inflamed analysis

TME analysis by xCell,^45^ CIBERSORT,^46^ Patient Immunophenoscore,^47^ quanTISeq,^48^ MCPcount,^49^ EPIC.^50^ A comprehensive immune‐related signature was constructed by concentrating on immune‐related genes obtained from ImmPort (https://immport.niaid.nih.gov), the list comprised a total of 2498 IRGs, covering 17 immune categories.^51^

### 2.12 Methylation

Download DNA methylation data of GBM patients using TCGAbiolinks in R. The methylation levels are stored in beta values ranging from 0 to 1. After removing data with missing values greater than 30%, perform an ANOVA test and extract MS‐specific methylation sites that show significant differences among the five subtypes.

### 2.13 Reverse‐phase protein array (RPPA) data analysis

RPPA measurements, where available, were used solely to corroborate pathway activation patterns inferred from transcriptomics. RPPA data were not used for clustering, feature selection, subtype assignment, model training, or threshold optimization. All subtype labels (C1–C5) were derived from the transcriptomic pipeline; RPPA results are presented descriptively to confirm directionality for selected markers and pathways.

### 2.14 Drug dataset

(1) Genomics of Drug Sensitivity in Cancer (GDSC, https://www.cancerrxgene.org): Cell lines are searched in GDSC to identify their sensitive drugs. There are 6768 treatment experiments for cell lines (*n =* 28) and drugs (*n =* 287), and 6380 treatment experiments when filtered by the AUC value (cut off by.7). Subtype‐specific analysis of drug sensitivity revealed a varied landscape: the C1 subtype was sensitive to 131 drugs, C2 to 109, C3 to 38, C4 to 79, and C5 to 133. We further probe into the intricacies of MSig subtype‐specific drug sensitivity, focusing on the analysis of cell lines, IC50 values, and targeted genes for drugs specific to each MSig subtype.

(2) PRISM database: The drug response data (IC50) for 1502 drugs was obtained from the PRISM database (secondary‐screen‐dose‐response‐curve‐parameters.csv) to match MS‐specific genes as a target‐drug dataset.^52^ Jurica et al.^53^ summarized mutational markers for drug sensitivity of cancer cells.

https://static‐content.springer.com/esm/art%3A10.1038%2Fs41467‐022‐305823/MediaObjects/41467_2022_30582_MOESM2_ESM.xlsx, combined SBS reposed for drug of statistically significant associations in the GDSC/PRISM “two‐way” replication test, GDSC/PSCORE “two‐way” replication test, and the GDSC/GDSC (same target) ‘two‐way’ replication test into the connection of SBS and drug dataset, which is the SBS‐drug dataset.

### 2.15 MSig‐specific features

Differences were compared using limma (gene expression), ANOVA (multi‐omics variance test), and *t*‐test (variance test between two groups), scheduled for *p* < .05 was the characteristic of a significant difference.

### 2.16 Prognosis analysis

Survival analysis was performed using the Kaplan–Meier test, and univariate Cox analysis and relative analysis were performed using the Pearson test.

### 2.17 Enrichment analysis

KEGG signalling pathways, and Gene Ontology (FDR < .05 as statistically significant by the R package *clusterProfiler*).

### 2.18 Correlation analysis

Correlation analysis was determined using Pearson correlation in R. In addition, *p*‐values for assessing the statistical significance of the correlation values were also calculated (*p* < .05).

## 3 RESULTS

### 3.1 Multi‐omics profile of GBM

**FIGURE 1:**
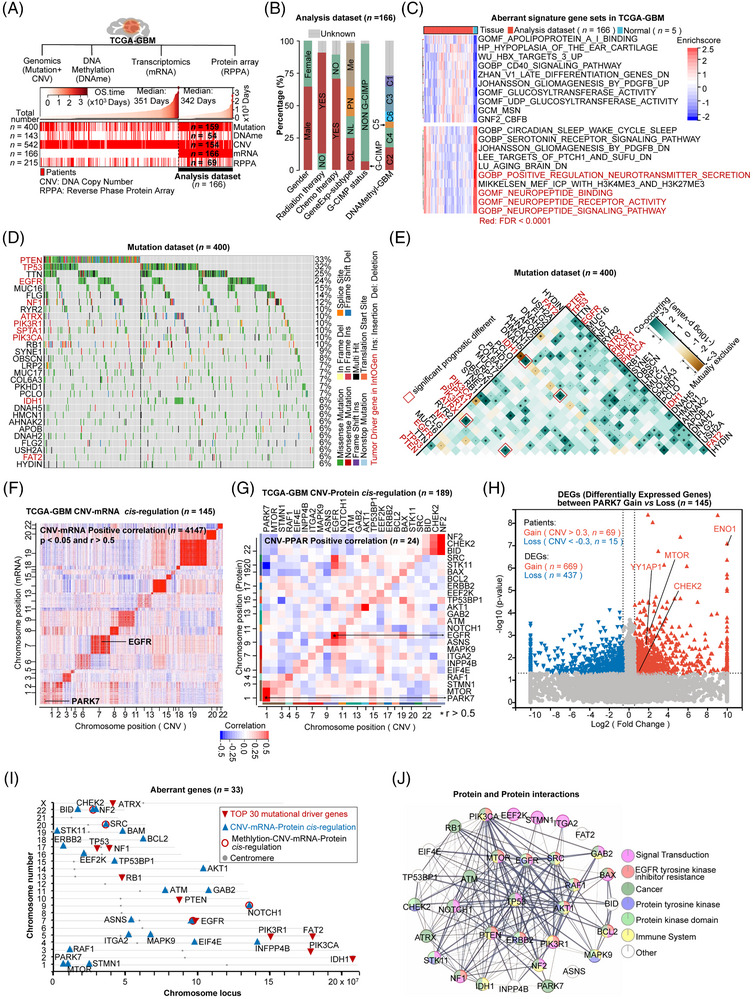
The multiple omics profile of GBM. (A) A schematic displaying the range of data types used in this study: WES (whole‐exome sequencing), CNA (copy number variation), DNAme (DNA methylation), and RPPA (reverse phase protein array). The analysis dataset, consisting of transcriptome data (*n =* 166), aligns with multiple omics data. In this schema, patient data are marked in red. OS (overall survival) is defined as the duration from diagnosis to death. (B) Stacked histograms showing the distribution of patient phenotypes (*n =* 166). This includes chemotherapy (temozolomide or anaplastic lymphoma kinase), radiation therapy, G‐CIMP status (cytosine‐phosphate‐guanine island methylator phenotype), and DNAmethyl‐GBM (DNA methylation subtypes, clusters C1–C6, as per Brennan et al.^6^). Percentages are detailed in the text. The rest of the description is clear and concise. (C) The top 20 significantly different molecular signatures (*p* < .05) were identified using MSig DB by comparing GBM (*n =* 166, red bar) to normal tissue (*n =* 5, light blue bar) using limma. Further details can be found in Table S2; red highlights FDR < .0001, indicating the highest significance. (D) Waterfall plots displaying the top 30 mutation genes in TCGA‐GBM (WES dataset: *n =* 400), with red marking tumour driver genes from IntOGen (Matthew H et al., 2018); different colours represent various mutation types. (E) Analysis of either mutually exclusive or co‐occurring top 30 mutation genes in TCGA‐GBM (WES dataset: *n =* 400) was conducted via pair‐wise Fisher's exact test to identify significant gene pairs (*p* < .05). Red boxes denote significant prognostic gene pairs (*p* < .05). (F) Heatmaps illustrating the correlation between copy number alterations (CNVs) and mRNA localized on chromosomes (*n =* 4147 pairs, *p* < .05, correlation *r* > .5). This demonstrates cis‐regulation: a positive correlation between CNV and corresponding mRNA expression. EGFR and PARK7 are specifically marked; patient count: *n = *145. (G) Heatmaps depicting correlations between CNVs and proteomics, showcasing CNV‐protein correlation pairs (*n =* 24, *p* < .05, *r* > .5). This reflects a positive correlation across CNV, mRNA, and protein expression; patient count: *n =* 189. (H) The Volcano map of DEGs (Differentially Expressed Genes) between patients with gain (CNV > .3, *n =* 69) vs. loss (CNV < −.3, *n =* 15) of PAPRK7 CNV. Red‐marked genes also exhibit differential expression of PARK7 protein in gain vs. loss groups. (I) Chromosome coordinate map of 33 aberrant GBM genes, including the top 10 in mutation rate and 24 with significant CNV‐mRNA‐Protein correlations. EGFR is unique in its top mutation rate and significant CNV–mRNA–protein correlations. (J) Protein–protein interaction (PPI) analysis of 33 aberrant GBM genes, with colours indicating involvement in various biological processes and pathways.

We utilized multi‐omics data from the Cancer Genome Atlas GBM (TCGA‐GBM) project dataset to map GBM heterogeneity. This included genomics (mutations: *n =* 400; DNA copy number variations, CNV: *n =* 542), DNA methylation (DNAme: *n =* 143), transcriptomics (mRNA: *n =* 166), and reverse phase protein array (RPPA: *n =* 215). We used patients with matched multi‐omics profiles (*n =* 166) as our analysis dataset (Figure 1A; Table S1). The composition of clinical features and molecular characteristics for the analysis dataset was characterized (Figure 1B; Table S1). Briefly, the clinical features from the Analysis dataset revealed: (1) the patient median survival is 342 days (*n =* 166), with the longest survival recorded at 3667 days; (2) it consists of 65.5% male and 34.5% female patients; (3) GBM clinical treatment includes 71% radiation, and 75% chemotherapy therapy (Table 1; Figure 1B). The molecular characterization features: (1) 34% of patients were mesenchymal (ME), 29% proneural (PL), 17% neural (NL), and 20% classical (CL) subtypes according to the gene expression (GeneExp‐) subtype;^5^ (2) the cytosine‐phosphate‐guanine (CpG) island methylator phenotype (G‐CIMP) was present in 11 out of 166 patients (Figure 1B; Table 1). Next, we employed the GSVA package to calculate patient enrichment scores for molecular signatures based on MSig DB gene sets, which are associated with biological processes, cellular functions, disease states, and tumour hallmarks.^24^ This process generated a GBM molecular signature matrix (MSig‐matrix) for 171 samples (166 GBM patients and 5 normal samples; *n =* 31 613 molecular signature sets) (Table S2). We compared the enrichment of molecular signatures between GBM patients and normal tissue (*p* < .01, fold change > |2|). Generally, we identified a significant reduction in neuro‐transduction pathways (Figure 1C, false discovery rate (FDR) < .0001).

**TABLE 1:** Baseline characteristics of the study cohort stratified by five molecular clusters.

Characteristics	C1 (*N* = 35)	C2 (*N* = 44)	C3 (*N* = 32)	C4 (*N* = 27)	C5 (*N* = 28)	Total (*N* = 166)	*p*‐value
OS time							<.01
Mean	488.51	422.86	464.38	361.37	380.14	427.50	
Median	406.00	417.00	338.50	269.00	358.50	360.00	
Gender							.99
Female	13(7.83%)	16(9.64%)	10(6.02%)	10(6.02%)	10(6.02%)	59(35.5%)	
Male	22(13.25%)	28(16.87%)	22(13.25%)	17(10.24%)	18(10.84%)	107(64.46%)	
Age							
Mean	61.79	56.03	61.18	62.25	59.76	59.94	
Median	62.60	58.58	61.55	62.52	61.31	60.80	
G‐CIMP status							<.001
G‐CIMP	0(0%)	9(5.56%)	1(0.62%)	0(0%)	1(0.62%)	11(6.79%)	
Non‐G‐CIMP	33(20.37%)	34(20.99%)	30(18.52%)	27(16.67%)	27(16.67%)	151(93.21%)	
GeneExp‐subtype							<.001
Classical	7(4.29%)	14(8.59%)	7(4.29%)	2(1.23%)	11(6.75%)	41(25.15%)	
Mesenchymal	8(4.91%)	4(2.45%)	12(7.36%)	22(13.50%)	10(6.13%)	56(34.36%)	
Neural	12(7.36%)	4(2.45%)	4(2.45%)	3(1.84%)	5(3.07%)	28(17.18%)	
Proneural	6(3.68%)	22(13.50%)	8(4.91%)	0(0%)	2(1.23%)	38(23.31%)	
DNA methyl							<.001
Cluster 1	2(1.65%)	1(0.83%)	3(2.48%)	8(6.61%)	0(0.0%)	14(11.57%)	
Cluster 2	7(5.79%)	3(2.48%)	7(5.79%)	5(4.13%)	7(5.79%)	29(23.97%)	
Cluster 3	7(5.79%)	5(4.13%)	8(6.61%)	4(3.31%)	8(6.61%)	32(26.45%)	
Cluster 4	5(4.13%)	11(9.09%)	0(0%)	0(0%)	9(7.44%)	25(20.66%)	
Cluster 5	0(0%)	7(5.79%)	0(0%)	0(0%)	1(0.83%)	8(6.61%)	
Cluster 6	0(0%)	8(6.61%)	4(3.31%)	0(0%)	1(0.83%)	13(10.74%)	
Chemotherapy							.4
No	16(9.64%)	14(8.43%)	9(5.42%)	9(5.42%)	5(3.01%)	53(31.93%)	
Yes	17(10.24%)	26(15.66%)	21(12.65%)	18(10.84%)	21(12.65%)	103(62.05%)	
Radiation therapy							.33
No	11(6.63%)	5(3.01%)	6(3.61%)	7(4.22%)	3(1.81%)	32(19.28%)	
Yes	22(13.25%)	35(21.08%)	24(14.46%)	20(12.05%)	23(13.86%)	124(74.70%)	

*Note*: Patient demographics, clinical features, and treatment patterns are summarized for the entire cohort (*N* = 166) and across the five molecular clusters (C1–C5). Continuous variables such as overall survival (OS) time (in days) and age (in years) are presented as mean and median. Categorical variables, including gender, G‐CIMP status, gene expression subtype, DNA methylation cluster, chemotherapy, and radiation therapy, are presented as counts with percentages. *p*‐values were calculated to test for differences across the five molecular clusters (C1–C5); overall survival (OS) time was compared using the log‐rank test; age was compared using one‐way ANOVA; all categorical variables were compared using the Chi‐square test.

Mutation analysis identifies the top 30 somatic alterations by analyzing genomic data (*n =* 400), such as the 33% mutation rate of PTEN, 32% of TP53, and 24% of EGFR (Figure 1D; Table S3), consistent with previous studies.^5^, ^6^ Other tumour driver genes (NF1, ATRX, PIK3R1, SPTA, and PIK3CA) marked by IntOGen,^54^ were also found among the top 12 mutated genes (Figure 1D). Co‐occurring mutations are simultaneous mutations in two or more genes within a tumour cell. Conversely, mutually exclusive mutations result from competitive selection, allowing only one mutation in a set of genes.^55^, ^56^, ^57^, ^58^, ^59^ We analyzed co‐occurrence and mutual exclusivity patterns. Mutations co‐occurring in TP53 and ATRX were associated with improved survival compared with either mutation alone (Kaplan–Meier, log‐rank *p* < .05), indicating a co‐mutation pattern facilitates tumour cell survival. The underlying mechanism remains to be determined^60^, ^61^ (Figure 1E; Figure S1A). However, PTEN and PIK3CA co‐occurrence did not show significant prognostic differences compared with single mutations (Figure S1B). The cis‐regulation analysis of alterations in copy‐number DNA segment variation (CNV) and its consequent mRNA/protein expression reveals the interconnected roles in driving tumour development.^62^, ^63^, ^64^, ^65^ For instance, IL‐18 amplification in CNV, which correlated with increased IL‐18 protein expression, plays a key modulatory role in the tumour microenvironment of non‐small‐cell lung cancer.^66^ We identified 4,147 cis‐regulations (*p* < .05 and *r* > .5) between CNV and mRNA expression, highlighting the dysregulation of biological pathways (Figure 1F; Figure S1D,E; Table S4) among the 9113 positive correlations (*p* < .05 and *r* > .0; Figure S1C). Cis‐regulation genes involving CNV, mRNA, and proteins (correlation across three omics levels, *p* < .05, *r* > .5; protein expression data from RPPA) were also identified, such as EGFR (*r* = .75) and PARK7 (*r* = .57) (Figure 1G; Table S4). Previously, EGFR has been reported as an important driver gene in glioblastoma multiforme (GBM), associated with aggressive tumour growth and therapeutic resistance.^67^ PARK7 plays a vital role in the oxidative stress response, protecting neurons from oxidative damage and cell death.^68^ Interestingly, we found that high expression of PARK7 in GBM significantly correlated with poorer patient survival and an increased 1.82‐fold risk of death compared with low PARK7 expression (cutoff: median FPKM; *p* < .0001) (Figure S1F–H). Differentially expressed genes (DEGs) between high and low PARK7 expression subgroups were enriched in upregulated cell cycle pathways (Figure S1I). Notably, we identified significant increases in mRNA and protein expression of oncogenes (ENO1, MTOR, YAPAP1, and HER2) in the high PARK7 expression subgroup (Figure 1H; Table S5). These findings highlight the potential for predicting patient prognosis. Next, we identified the chromosomal locations of the top ten tumour driver and cis‐regulation genes (Figure 1I). Notably, EGFR was found to be aberrantly regulated across multiple omics, including activating mutations, methylation, and copy number variations. This led to high expression at both mRNA and protein levels (Figure 1I). Furthermore, analysis of EGFR's protein‐protein interaction (PPI) network^69^ revealed associations with other kinase activities and the immune system (Figure 1J).

### 3.2 The mutational signature landscape of GBM reveals environmental factors

**FIGURE 2:**
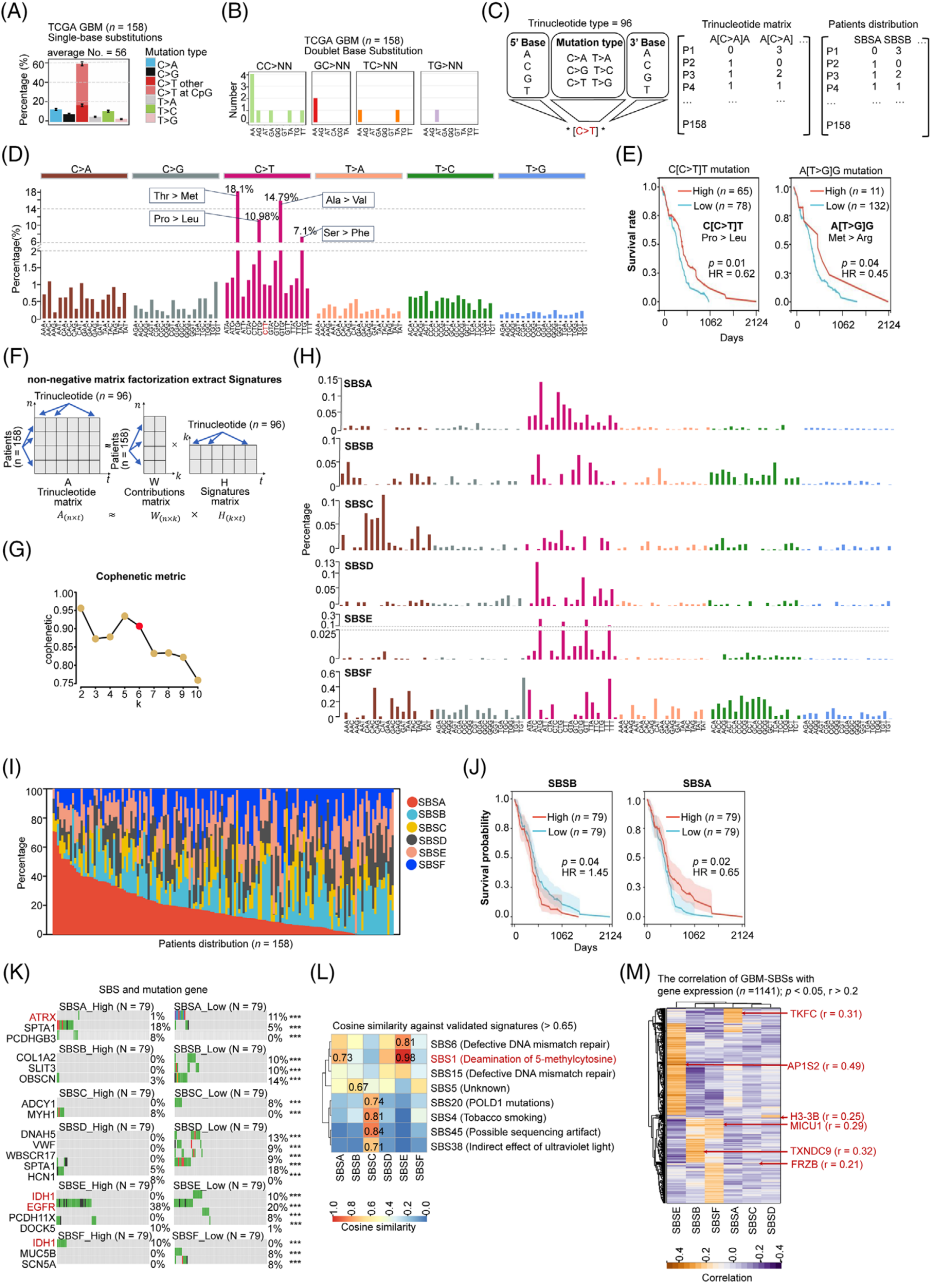
The mutational signature landscape of GBM reveals environmental factors. (A) The percentage distribution of single‐base substitutions in GBM patients (*n =* 158). (B) The count of doublet base substitutions (DBS) observed in GBM patients (*n =* 158). (C) Analysis of trinucleotide patterns (*n =* 96), including a trinucleotide matrix and patient distribution for Figure 2F. (D) The percentages of 96 trinucleotide substitutions in GBM patients (*n =* 158). The bottom section lists the 96 replaced trinucleotides, with the top four types highlighted (e.g., threonine [Thr], methionine [Met], proline [Pro], and leucine [Leu]). (E) Kaplan–Meier survival analysis of patients categorized by high and low percentages of specific trinucleotide substitutions (C[C > T]T and A[T > G]G). Significance is determined using the log‐rank test, with cut‐offs established via *maxstat* in R. (F) Application of non‐negative matrix factorization (NMF) to derive new mutational signatures and patient contributions based on the trinucleotide matrix. (G) Presentation of the Cophenetic metric from NMF, where *k* denotes the signature number. The red point indicates the optimal signature number. (H) Identification of six new mutational signatures (SBSA, SBSB, SBSC, SBSD, SBSE, SBSF) derived from NMF of TCGA‐GBM. The lower section shows the 96 replaced trinucleotides. (I) The percentage distribution of the six newly identified mutational signatures across patients (*n =* 158). (J) Kaplan–Meier survival analysis of patients based on the percentages of SBSB and SBSA, with high and low percentage cut‐offs determined by the median. (K) Patients were divided according to the median distribution of each GBM‐SBS, and a subsequent comparison of mutation genes in groups with high and low SBSs. ***p < .001. (L) Evaluation of the relationship of the six GBM‐SBSs with previously reported 30 single‐base substitutions (SBSs, as described by Sondka et al.^41^), which account for tumour initiator. (M) Heatmap illustrating the correlation analysis between GBM‐SBSs and gene expression.

Genomic fingerprints of mutational signatures have been previously used to identify potential triggering initiators (such as UV light exposure, smoking, etc.^70^) and causal treatment responses (e.g., BRCA mutation‐associated signatures often responding to PARP inhibitors^71^). We further analyzed substitution patterns in mutational signatures to identify exposure‐mediated triggers. We examined somatic substitution mutations in GBM WES data (*n =* 158) using *maftools* ^38^ and found an average of 56 single‐base substitutions (SBS) per patient, with C > T mutations constituting 58% (Figure 2A). Doublet base substitutions (DBS), where two adjacent DNA bases are simultaneously substituted, were identified in 12 patients, most frequently as CC > NN type (*n =* 7) (Figure 2B). Survival analysis did not reveal a significant difference between patients with and without DBS (Figure S2A). We further analyzed mutational signatures based on single‐base substitutions within trinucleotides. The most common types were A[C > T]G (18.1%, threonine to methionine), G[C > T]G (14.97%, alanine to valine), C[C > T]G (10.98%, proline to leucine), and T[C > T]G (7.1%, serine to phenylalanine) (Figure 2C,D; Table S6). Importantly, trinucleotide substitutions C[C > T]T (1.53%) and A[T > G]G (<.13%) were significantly associated with patient prognosis (Figure 2E).

Second, we applied non‐negative matrix factorization (NMF) to the 96‐mutational matrix (representing patient samples as rows and trinucleotide contexts as columns; Figure 2C,F; Table S7). We identified six novel mutational signatures, named GBM‐SBSs (SBSA to SBSF) (Figure 2F–G), and analyzed their distribution across patients (Figure 2I). Notably, SBSA and SBSB were significantly associated with prognosis: higher levels of SBSA correlated with increased survival rates, while higher levels of SBSB were linked to decreased survival rates. This association was determined using a cutoff based on the median SBSs distribution (hazard ratio = 1.45, *p* = .04) (Figure 2J). Furthermore, we divided patients into high and low GBM‐SBS groups based on the median distribution to examine mutational preferences: (1) 18% of patients with high SBSA distributions carried SPTA1 mutations, while 11% of patients with low SBSA had ATRX mutations; (2) low SBSB patients exhibited mutations in COL1A2 (10%), SLIT3 (10%), and OBSCN (14%); (3) high SBSC patients had MYH1 mutations (8%), while low SBSC patients had ADCY1 mutations (8%); (4) high SBSE patients had EGFR (38%) and DOCK5 (10%) mutations; and (5) all IDH mutations (10%) were exclusively present in low SBSE and high SBSF patients (Figure 2K).

Next, we compared the six GBM‐SBSs with the previously reported 30 SBSs (Catalogue of Somatic Mutations in Cancer, COSMIC v3.2) using cosine similarity as described in previous studies.^40^, ^41^ These analyses revealed environmental factors influencing the mutational landscape of GBM: (1) GBM‐SBSA and SBSE closely matched COSMIC‐SBS1 (cosine‐similarity:.73 and.98, respectively), suggesting that patients with high SBSA and SBSB undergo spontaneous or enzymatic deamination of 5‐methylcytosine; (2) GBM‐SBSF closely matches COSMIC‐SBS6 (cosine‐similarity:.81), which has been previously reported to be associated with defective DNA mismatch repair; (3) GBM‐SBSC closely matches COSMIC‐SBS4 (cosine‐similarity:.81), indicating that patients carrying high SBSC are associated with smoking exposure (Figure 2L). Additionally, we analyzed the impact of GBM‐SBSs on transcriptome‐wide gene expression through correlation analysis and identified 1141 genes with strong positive correlations to GBM‐SBS distributions (*p* < .05, *r* > .2) (Figure 2M; Table S8). The strongest correlations between gene expression and GBM‐SBSs include TKFC with SBSA (*r* = .31), TXNDC9 with SBSB (*r* = .32), FRZB with SBSC (*r* = .21), H3‐3B with SBSD (*r* = .25), AP1S2 with SBSE (*r* = .49), and MICU1 with SBSF (*r* = .29) (Figure 2M). KEGG pathway enrichment analysis revealed that genes positively correlated with SBSA are significantly enriched in metabolic pathways, while those correlated with SBSB are associated with aberrant ribosome pathways (Figure S2B–E).

### 3.3 Identification of prognostically relevant GBM subtypes by integrated molecular signatures

**FIGURE 3:**
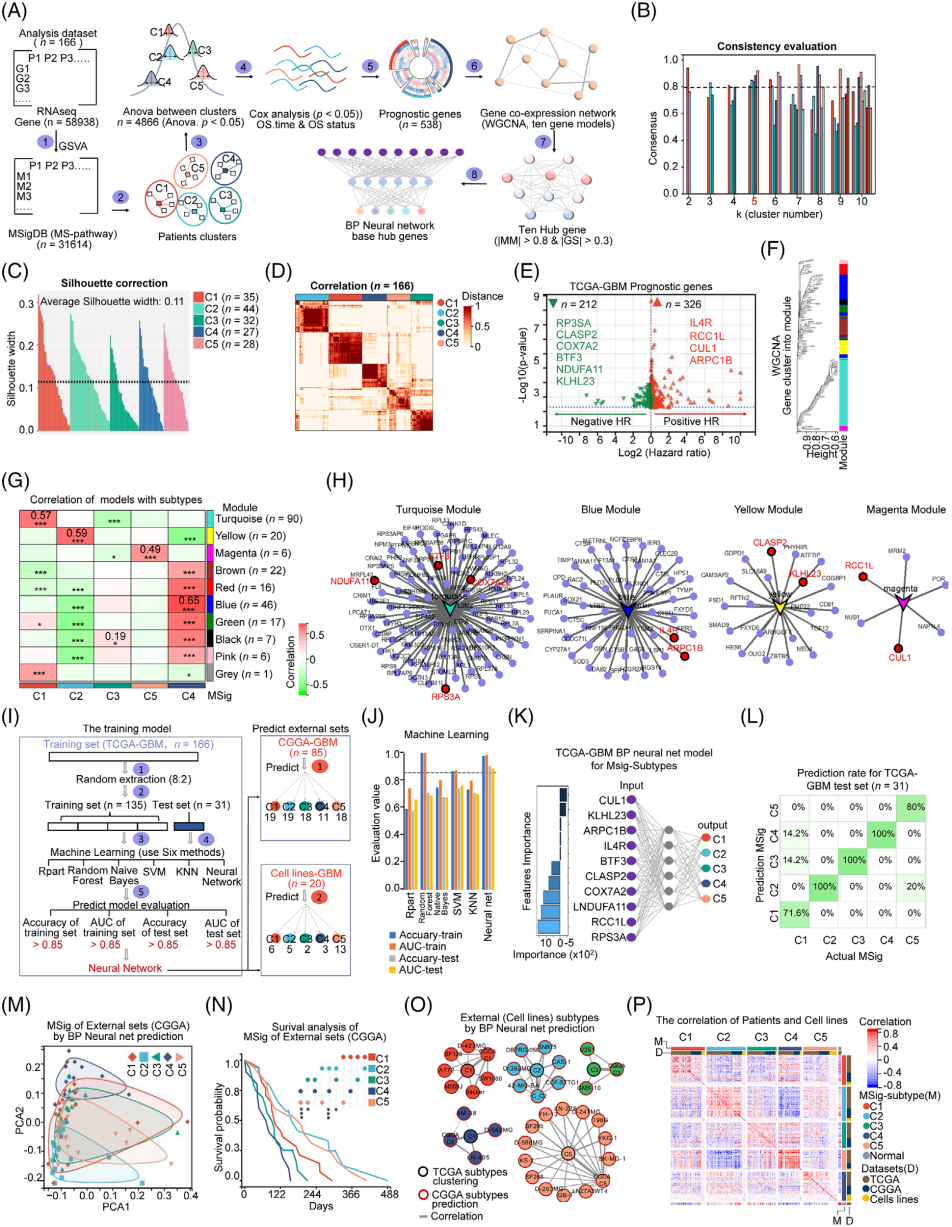
Identification of prognostically relevant GBM subtypes by integrated molecular signatures. (A) Outline of the workflow for clustering and prediction processes. (B) Assessment of consistency in sample clustering for 2–10 clusters, highlighting the optimal cluster number (*k* = 5). (C) Evaluation and refinement of silhouette coefficients to identify five GBM MSig (molecular signature) subtypes. (D) Heatmap representation of patients across the five MSig subtypes of TCGA‐GBM, including corrections in gene model and MSig subtypes. Significance levels range from ****p < .0001 to *p < .05. (E) Prognostic gene analysis in TCGA‐GBM via Cox analysis of 4866 significantly differentially expressed genes across the five MSig subtypes. Red indicates high gene expression correlating with a positive prognosis (positive HR, *n =* 326), while green represents high gene expression associated with a negative prognosis (negative HR, *n =* 212). (F, G) WGCNA segmentation of gene expression into ten modules, with each vertical colour representing a distinct module. Correlation analysis highlights four modules most strongly correlated among the five subtypes: turquoise, yellow, blue, and magenta. (H) Identification of representative genes in significant co‐expression modules (turquoise, yellow, blue, and magenta) through WGCNA. Red indicates hub genes with high gene significance (|GS| > .3) and module membership (|MM| > .8). (I) Workflow for developing an AI model to predict new datasets (CGGA‐GBM and GBM cell lines) of GBM. (J) Evaluation of six machine learning models based on AUC and Accuracy in training (*n =* 135) and test datasets (*n =* 21), with a patient cutoff ratio of 8:2. Detailed evaluation values are provided in Table S11. (K) Description of the BP neural net model for TCGA‐GBM MSig subtypes, showing gene feature importance on the left and model architecture (number of layers, *n =* 1; hidden neurons, *n =* 6) on the right. (L) Prediction rate for the TCGA‐GBM test dataset (*n =* 31) using the BP neural net model. (M) PCA analysis of five MSig subtypes predicted by the BP neural net model in the external CGGA‐GBM dataset. (N) Kaplan–Meier survival analysis of patients across five MSig subtypes in the external CGGA‐GBM dataset, with significance determined by a Log‐rank test (**p < .001, *p < .05). (O) Prediction of MSig subtypes in 30 GBM cell lines using the BP neural network model. (P) Heatmap showing the correlation among TCGA‐GBM patients, CGGA‐GBM patients, and cell lines based on overlapping gene expression; M indicates MSig subtypes, and D represents datasets.

To identify clinically relevant GBM subtypes, we performed pathway‐based classification using unsupervised clustering. We first compute patient‐level pathway activity (GSVA^37^) and cluster these activities; we then integrate complementary omics layers to anchor each subtype mechanistically and assess clinical relevance (Figure 3A; Table S2). We identified an optimal cluster number of *k* = 5 (five clusters, with a mean consensus value of clusters over.85) using Consensus Clustering^42^ based on the MSig‐matrix, which showed the minimal impact from random sampling (Figure 3B; Table S2). We assessed intercluster distances using the Silhouette metric^72^ and adjusted them to achieve five distinct subtypes: C1 (*n =* 35), C2 (*n =* 44), C3 (*n =* 32), C4 (*n =* 27), and C5 (*n =* 28) (Figure 3C–D; Table S1). Using the Jaccard = .6 filter, we retained 803 signatures. The five‐cluster solution remained stable when deconvoluted using either Hallmark‐only or KEGG/Reactome‐only gene sets, with global adjusted rand index (ARI) values of.15 for both collections. Crucially, each subtype was highly distinct in a one‐versus‐rest analysis (Hallmark‐only: C1 = .91, C2 = .86, C3 = .89, C4 = .81, C5 = .91; KEGG/Reactome‐only: C1 = .89, C2 = .86, C3 = .88, C4 = .89, C5 = .87), confirming their unique identities. We further compared the predicted patient prognosis from previously classified GBM subtypes (GeneExp, IDH mutation, methylation, and MSig subtypes) (Figure S3A–D). These analyses highlighted the strong correlation of MSig subtypes in molecular characteristics with patient prognosis.

Second, to identify the MSig‐biomarker, we performed: (1) screening significantly differentially expressed genes across the five MSig subtypes (*n =* 4866, ANOVA *p* < .05); (2) selecting 538 prognostic genes through univariate Cox regression analysis (Figure 3E); (3) grouping the prognostic genes (*n =* 538) into co‐expression modules, and identified ten Hub genes using WGCNA^43^ (Figure 3F–H; Tables S9 and S10). Specifically, we identified four WGCNA co‐expression modules (turquoise, yellow, blue, and magenta) showing the highest significant correlations with the five MSig subtypes (Figure 3G). Hub genes from WGCNA are crucial in regulating their respective co‐expression networks.^43^ We identified the top ten prognostic hub genes for each MSig subtype (GS: the correlation between the node and the phenotype, |GS| > .3) and module membership (|MM| > .8), pinpointing RPS3A and NDUFA11 for the C1 subtype in the turquoise, CLASP2, and KLHL23 for the C2 subtype in Yellow, BTF3 and COX7A2L for C3 in turquoise, IL4R and ARPC1B for C4 in blue, and RCC1L and CUL1 for C5 in magenta (Figure 3H; Figure S3E–G).

Next, we applied classified algorithms (Rpart, Random Forest, Naive Bayes, SVM, KNN, and Neural Net) to build machine learning prediction models for hub gene prediction (*n =* 166, training:test = 8:2) (Figure 3I). The BP neural network is superior to the other algorithms with an AUC of.89 and an accuracy of.9, using a single hidden layer with six neurons (Figure 3J–K; Table S11). We validated the BP neural network predictive performance on the test dataset (*n =* 31) (Figure 3L), external patients in CGGA‐GBM (*n =* 85) and GBM cell lines (*n =* 30) to predict MSig subtypes (Figure 3M,O; Table S12). PCA of CGGA‐GBM MSig subtypes demonstrated the model's effectiveness (Figure 3M). Survival analysis in the CGGA‐GBM confirmed the poor prognosis of the C4 subtype (Figure 3N), which is consistent with the survival rate of the TCGA dataset (Figure S3D). We also showed that the predicted MSig classification in GBM cell lines is consistent across TCGA‐GBM, CGGA‐GBM subtypes (Figure 3O,P; Table S12). The model achieved an overall F1 of.80 on the CGGA external hold‐out and.83 under fivefold cross‐validation on TCGA; per‐class F1‐scores (C1–C5) and macro/weighted averages are reported in Table S11. Overall, our BP neural network with a ten‐hub gene predicted model is effective in predicting MSig‐subtypes in external datasets of GBM. The time‐dependent ROC analysis revealed C4's superior prognostic performance (1‐year AUC = .76), significantly outperforming other subtypes (DeLong's test *p *< .01). This aligns with its distinct immune‐exhausted phenotype and supports clinical prognosis classification.

### 3.4 Mutation landscape of GBM MSig subtypes

**FIGURE 4:**
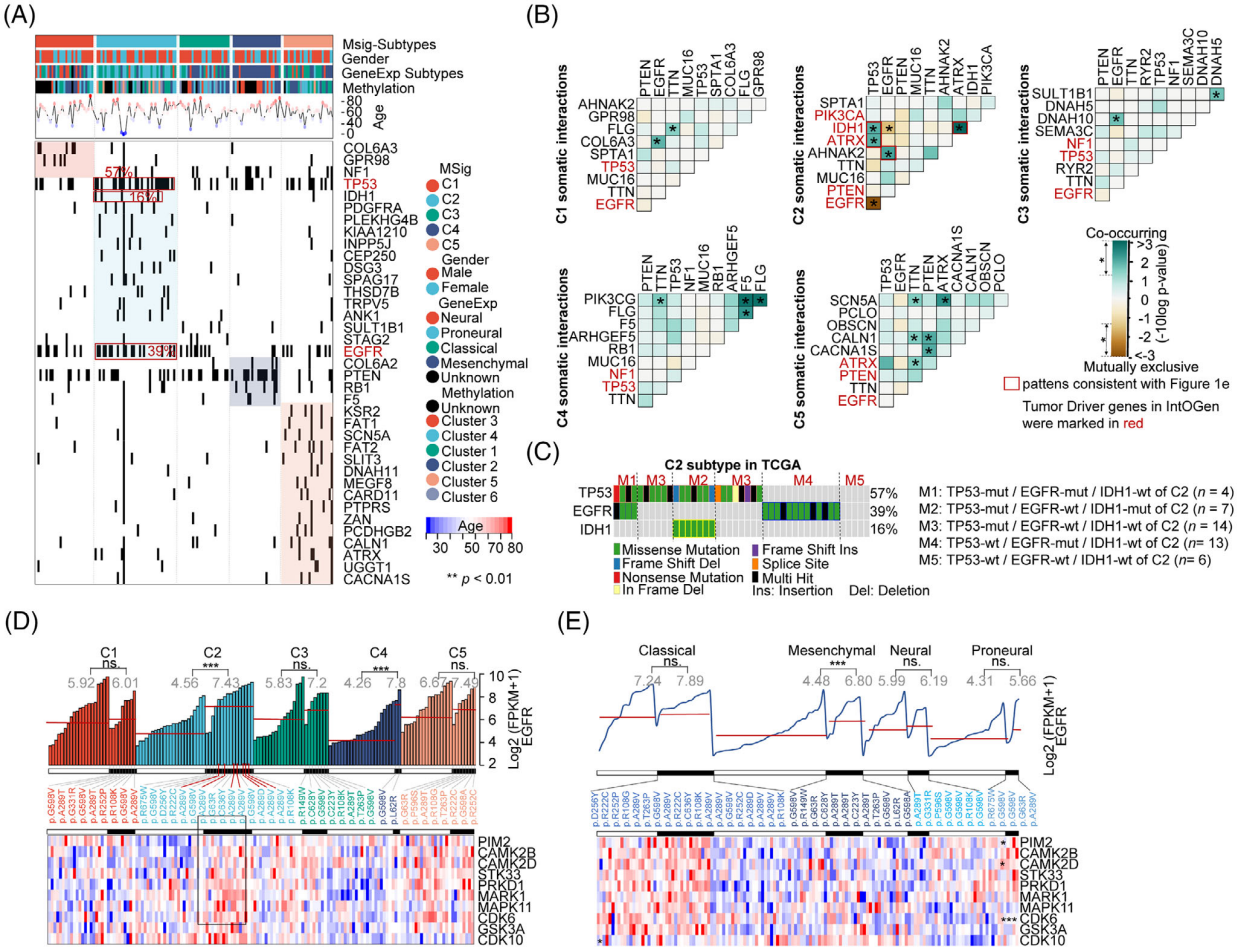
Mutation landscape of GBM MSig subtypes. (A) Analysis of 37 mutation genes displaying differential distribution patterns across five MSig GBM subtypes. The focus is on DNA methylation subtypes (as described in Brennan et al.^6^), with TP53, EGFR, and IDH1 mutations predominantly observed in the C2 subtype. Significance was determined by ANOVA analysis: **p < .01. (B) Exploration of co‐occurrence and mutual exclusivity in mutation patterns across five MSig GBM subtypes. Tumour driver genes from IntOGen are marked in red, with red boxes indicating patterns consistent with the overall GBM signature (*p < .05). (C) Classification of patients within the C2 subtype based on TP53, EGFR, and IDH1 mutations into five groups (M1–M5). (D, E) Examination of EGFR mutations (including mutation sites), gene expression, and correlations with the top ten genes across five MSig subtypes and four GeneExp subtypes. Patients with EGFR mutations are marked with a black bar; the red line represents the median EGFR expression in each MSig subtype. Statistical analysis was conducted via *t*‐tests: ***p < .001, ns = no significance.

The correlation between highly mutated tumour‐driver genes, such as PTEN (33%) and EGFR (24%), and GBM patient prognosis remains unclear.^73^ Next, we focused on the mutation landscape of GBM MSig subtypes. First, we compared the mutation profiles between GeneExp and MSig subtypes. We found (1) only three genes (PTEN, TP53, and TTN) consistently appeared in the top 10 mutations across all subtypes; the MSig‐C4 subtype exhibited the highest PTEN mutation rate (54%), followed by C1 (34%) and C2 (25%). (2) GeneExp‐ and MSig‐subtypes showed mutational profile similarities, sharing seven mutated genes between the MSig‐C1 and GeneExp‐neural subtypes, as well as six between MSig‐C2 and GeneExp‐proneural. (3) IDH1 was predominantly present in the MSig‐C2 (16%) and GeneExp‐proneural (20%) subtypes (Figure S4A,B). Second, we performed statistical analysis for each mutated gene (*n =* 14 073) across five MSig subtypes (*p*‐value of ANOVA across five subtypes < .05 and *p*‐value between two subtypes < .05; Table S13). We identified the top 37 significantly different mutational genes across the five MSig GBM subtypes (Figure 4A). Notably, the C2 subtype showed the highest mutation rates for certain genes: IDH1 (16% in C2, representing 88% of patients among all GBM cases), TP53 (57%, corresponding to 45% in all GBM), and EGFR (39%, corresponding to 36% in all GBM) (Figure 4A). Additionally, the C2 subtype exhibited the highest base substitutions per patient (*n =* 86), suggesting genomic instability (Figure S4D). Other mutated genes, including KSR2, SCN5A, FAT1, and FAT2, were predominantly found in the C5 subtype (Figure 4A).

Next, we examined the correlation of mutations and prognosis among GBM MSig subtypes. By comparing GeneExp and MSig subtypes, we identified the following key findings: (1) Mutational patterns across subtypes: The GeneExp‐Proneural subtype and MSig‐C2 both exhibit co‐occurring IDH1 and TP53 mutations, as well as ATRX and TP53 mutations. Gene‐Exp Neural and MSig‐C1 share the co‐occurring pair of EGFR and COL6A3 (Figure S4C,B). (2) Unique mutations in each subtype: We identified mutation patterns within the top nine mutational genes from each MSig subtype: C1 with 2 mutations, C2 with 6 mutations, C3 with 2 mutations, C4 with 4 mutations, and C5 with 6 mutations (*p* < .05) (Figure 4B). Notably, the C2 subtype uniquely defined four pairs of co‐occurring mutations: IDH1 and ATRX, IDH1 and TP53, ATRX and TP53, as well as EGFR and AHNAK2 (Figure 4B). (3) Prognostic significance: Mutations in TP53, EGFR, and IDH1 within the MSig‐C2 subtype were associated with significant prognostic differences (Figure S4E). In the GeneExp‐Proneural subtype, mutations of EGFR and IDH1 were found to have independent prognostic significance, while TP53 did not exhibit such associations (Figure S4F,G). Mutations in PTEN, TTN, and MUC16, the top 10 genes associated with GBM, did not show a significant impact on survival (Figure S4E). (4) Subclassification of C2 subtype patients: The C2 subtype patients were divided into five subcategories based on the mutual exclusivity between TP53 and EGFR mutations, as well as co‐occurrence with IDH1 mutations (Figure 4B,C). Interestingly, the M2 genotype (where TP53 is mutated, EGFR is wild‐type, and IDH1 is mutated) exhibited the best prognosis (Figure S4H), suggesting a protective role of IDH1 mutations. In contrast, the M4 genotype (with TP53 wild‐type, EGFR mutated, and IDH1 wild‐type) showed the worst prognosis (Figure S4H). Additionally, analysis of EGFR transcriptional expression across MSig subtypes revealed that EGFR mutations predominantly induce its overexpression in C2 subtypes (Figure 4D). This was positively correlated with C2 patient survival, emphasizing the role of EGFR mutational status (Figure S4E). A specific mutation site at amino acid 289 (P.A289_V/D) accounts for 44% of all EGFR mutation sites in C2 (*n =* 7), where higher mRNA expression (FPKM > 7.43) was observed (Figure 4D). However, this pattern of EGFR activation did not correlate with GeneExp‐subtype classification (Figure 4E).

### 3.5 Gene expression hallmarks of GBM MSig subtypes

**FIGURE 5:**
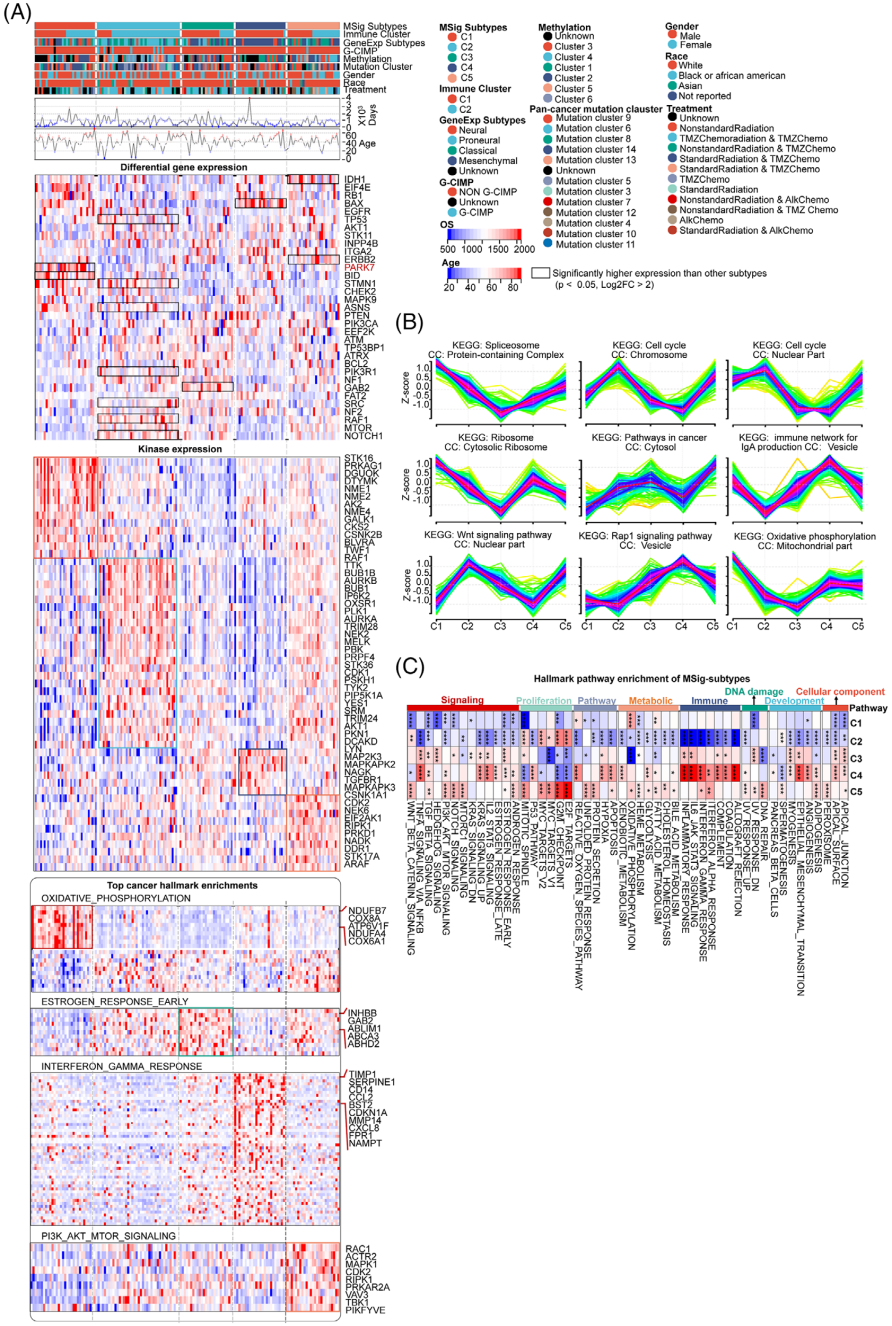
Gene expression hallmarks of GBM MSig subtypes. (A) Mapping of patients’ clinical features (including CIMP [cytosine‐phosphate‐guanine island methylator phenotype] and pan‐cancer mutation cluster status) was performed using data sourced from the UCSC Cancer Genomics Browser. Following this, the expression patterns of 33 abnormal molecular markers (as identified in Figure 1I) were assessed across the five MSig subtypes. A subset of 13 genes was found to overlap both the abnormal molecular markers and the CMCs list, showing significant variation in their distribution across the subtypes. Moving on to the distribution of 54 tumour‐related kinases from the CMCs list was analyzed across different MSig subtypes. Finally, detailed enrichment analyses of hallmark genes in subtypes C1, C3, C4, and C5 are presented, with *t*‐tests used to compare expression levels against other patients (significance marked as ****p < .0001). (B) Gene expression levels were analyzed across the five subtypes, with consistent trends observed. KEGG and GO enrichment analyses of these pathways are presented, along with an evaluation of cell components (CC). The analysis utilized a gene set comprising 5421 genes from the cancer molecular characteristics (CMCs) database, with specific counts provided for each MSig subtype. (C) Enrichment scores for tumour hallmarks were compared between the five MSig subtypes and normal tissue, highlighting the distinct oncogenic features acquired by human tumours. Statistical analyses, including *t*‐tests for pairwise comparisons, revealed significant differences (****p < .0001).

To further identify differential gene expression across MSig subtypes, we analyzed the differential distribution of tumour‐related genes (ANOVA *p* < .05, *n =* 157) from Positional Gene Sets (*n =* 301). We found aberrant gene expression in MSig‐specific chromosome locations, evidenced by increased gene counts in chromosomes 6, 16, 17, and 19 for C2, and chromosomes 7, 8, 13, and 21 for C4 subtypes (Table S14; Figure S5A). We established a list of differentially expressed genes (DEGs) for each subtype by comparing within TCGA‐GBM rest samples (*n =* 166, cutoff: *p* < .05 and Log2FC > 2) [C1: 1668 genes, C2: 1964 genes, C3: 208 genes, C4: 1,155 genes, C5: 426 genes; Table S15] and performed the following analyses. (1) We clustered these DEGs into 9 groups by *Mfuzz*,^74^ confirming subtype‐specific differences in gene expression. We further performed KEGG and GO enrichment analyses to show the status of nine biological pathways in each MSig subtype. Specifically, C1 exhibited higher enrichment in spliceosome, ribosome, and oxidative phosphorylation pathways; C2 showed enrichment in cell cycle and Wnt signalling pathways; and C4 was enriched in the immune network for IgA production and the Rap1 signalling pathway (Figure 5B). (2) 13 DEGs from previously identified abnormal molecular markers (Figure 1I) were also listed in Figure 5C. Notably, PARK7 (correlated with worst patient survival; Figure S1H) showed significantly high expression in C1 (log2FC: 38.78; Figure 5A; Table S15). Potential biomarkers for MSig subtypes were also identified, including STMN1 (log2FC: 27.15) and NOTCH1 (log2FC: 10.14) in C2, GAB2 (log2FC: 3.46) in C3, BAX (log2FC: 6.80) in C4, and IDH1 (log2FC: 11.18) in C5 (Figure 5A; Table S15). (3) The DEGs list includes 54 tumour‐related kinases, showing: C5 includes more highly expressed genes compared with other subtypes; C2 highly expressed in IP6K2, OXSR1, STK36, SRM; C1 in NME1, NME2, NME4; C4 in MAPK (MAP2K3, MAPKAPK2, MAPKAPK3) kinases (Figure 5A).

Moreover, we characterized cancer hallmarks^75^, ^76^ across MSig subtypes by the GSVA algorithm.^37^ This analysis allowed us to categorize the heterogeneous tumour characteristics (e.g., signalling pathways, proliferation/metabolic status, immune response). We evaluated hallmark enrichment across MSig subtypes and found: (1) C2 and C4 generally exhibited opposite hallmarks in many aspects, such as in the immune category; (2) C5 showed significant enrichment in tumour‐driving signalling, such as PI3K/ATK/MTOR and NOTCH pathways; (3) C3 was identified as a transitional state (Figure 5C; Table S16). Furthermore, we determined the top hallmark enrichments in each MSig subtype and marked the key genes as subtype‐specific biomarkers (Figure 5A). For example, C1 exhibited significant activation of OXIDATIVE_PHOSPHORYLATION within the metabolic category, involving key genes such as NDUFB7, COX8A, ATP6V1F, NDUFA4, and COX6A1 (Figure 5A).

To further validate our transcriptome‐derived subtypes, we applied the 10‐gene BP neural network classifier to the CPTAC GBM proteogenomic cohort (*n* = 110) prediction, which was consistent with TCGA‐based labels, and Hallmark pathway analysis on proteomic profiles confirmed subtype‐specific biology. For example, C4 tumours displayed elevated interferon‐γ response, EMT, and inflammatory pathways, whereas C2 tumours were enriched for cell cycle and DNA repair programs. C3 tumours retained globally low pathway activity, consistent with their transitional phenotype (Figure S5B). These findings demonstrate that the MSig subtypes are robustly detectable across independent transcriptomic and proteomic datasets.

### 3.6 Reconstruction of tumour pseudo‐time evolution of MSig GBM subtypes

**FIGURE 6:**
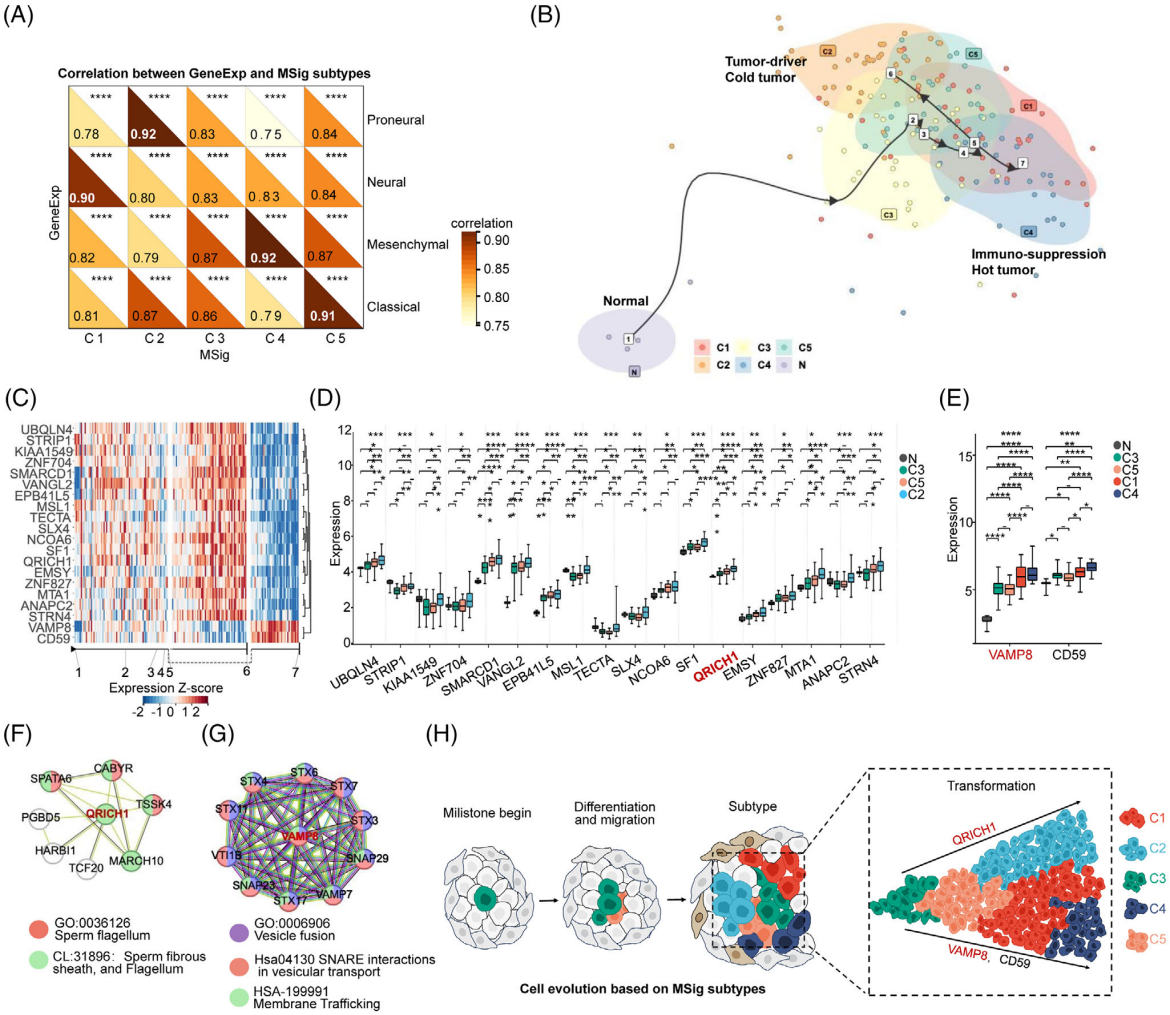
Reconstruction of tumour pseudo‐time evolution of MSig GBM subtypes. (A) A correlation analysis was conducted between GeneExp‐MSig subtypes based on all‐gene expression. Significant correlations were denoted as ***p < .05. (B) Using the dyno package in R, inferred tumour developmental trajectories for GBM and normal tissue were identified. These trajectories are highlighted by subtype (C1, C2, C3, C4, C5), with black lines indicating the inferred pathways, numbered from 1 to 7. (C) A heatmap was generated to illustrate the expression of branch node genes corresponding to the inferred trajectories (numbered from 1 to 7). (D‐E) The distribution of branch node gene expressions across five MSig subtypes was analyzed. Significant differences were observed at various levels (*p < .05 to ****p < .0001); a ‘‐’ indicates no significant difference. Notably, QRICH1 exhibited significant variation among normal tissue, C3, C5, and C2 subtypes. (F, G) Protein–protein interaction networks were analyzed using String (https://string‐db.org) for branch node genes QRICH1 and VAMP8. (H) An analysis of cell differentiation across the five MSig subtypes revealed that subtype C3 is the least differentiated.

We analyzed the transcriptome to map the trajectory of tumour progression. We carried out a correlation analysis between MSig and GeneExp‐subtypes within the transcriptome. Remarkably, C1 closely matched the GeneExp‐neural subtype (*r* = .90, *p* < .001), while C2 aligned with GeneExp‐proneural, C4 with GeneExp‐mesenchymal, and C5 with GeneExp‐classical subtypes (*r* values range from.91 to.92, *p* < .001 for all) (Figure 6A). Next, we performed pseudo‐time analysis^44^, ^76^, ^77^, ^78^ to build a trajectory based on the biological characteristics of GBM MSig subtypes. We mapped the evolutionary positions for each GBM patient, identified the tumour's invasive evolutionary pathways (trajectories 1–7) by using *Dyno*,^44^ and labeled them according to the five MSig subtypes of GBM patients (Figure 6B). Normal tissues (*n =* 5) were positioned at trajectory 1. The GBM began at trajectories 2 and 3 (C3 subtype), followed by trajectories 4–6 (C2 subtype), and extended to the other end of trajectories 4–7 (C4 subtype) (Figure 6B). This pattern is consistent with the results of the cancer hallmark analysis (Figure 5A). The GBM trajectory suggests that the lower evolutionary stage C3 subtype has two potential pathways towards either C2 or C4 endpoints. Next, we further analyzed the branch node genes that potentially determine cellular fate during disease progression.^44^ Top 20 branch node genes were identified, which is consistent with developmental trends across trajectories numbered 1 to 6 (Figure 6C). Among these, 18 genes displayed high expression in the C2 subtype, followed by the C3 and C5 subtypes (Figure 6C,D). QRICH1 showed significant expression differences among the C3, C5, and C2 subtypes (Figure 6D). Conversely, VAMP8 and CD59 were significantly highly expressed in the C4 subtype (trajectories numbered 1, 2, 3, 4, 5, and 7) (Figure 6E). QRICH1 was found to regulate processes associated with the sperm fibrous sheath and flagellum in cellular structural components (Figure 6F). Conversely, VAMP8 was linked to SNARE interactions in vesicular transport, membrane trafficking, and vesicle fusion (Figure 6G). We hypothesize that the expression patterns of QRICH1 and VAMP8 contribute to defining the trajectory trail for MSig GBM subtypes, offering insights into the tumour's evolutionary driving forces (Figure 6H).

### 3.7 Identification of ‘cold’ and ‘hot’ immunophenotypes of GBM MSig subtypes

**FIGURE 7:**
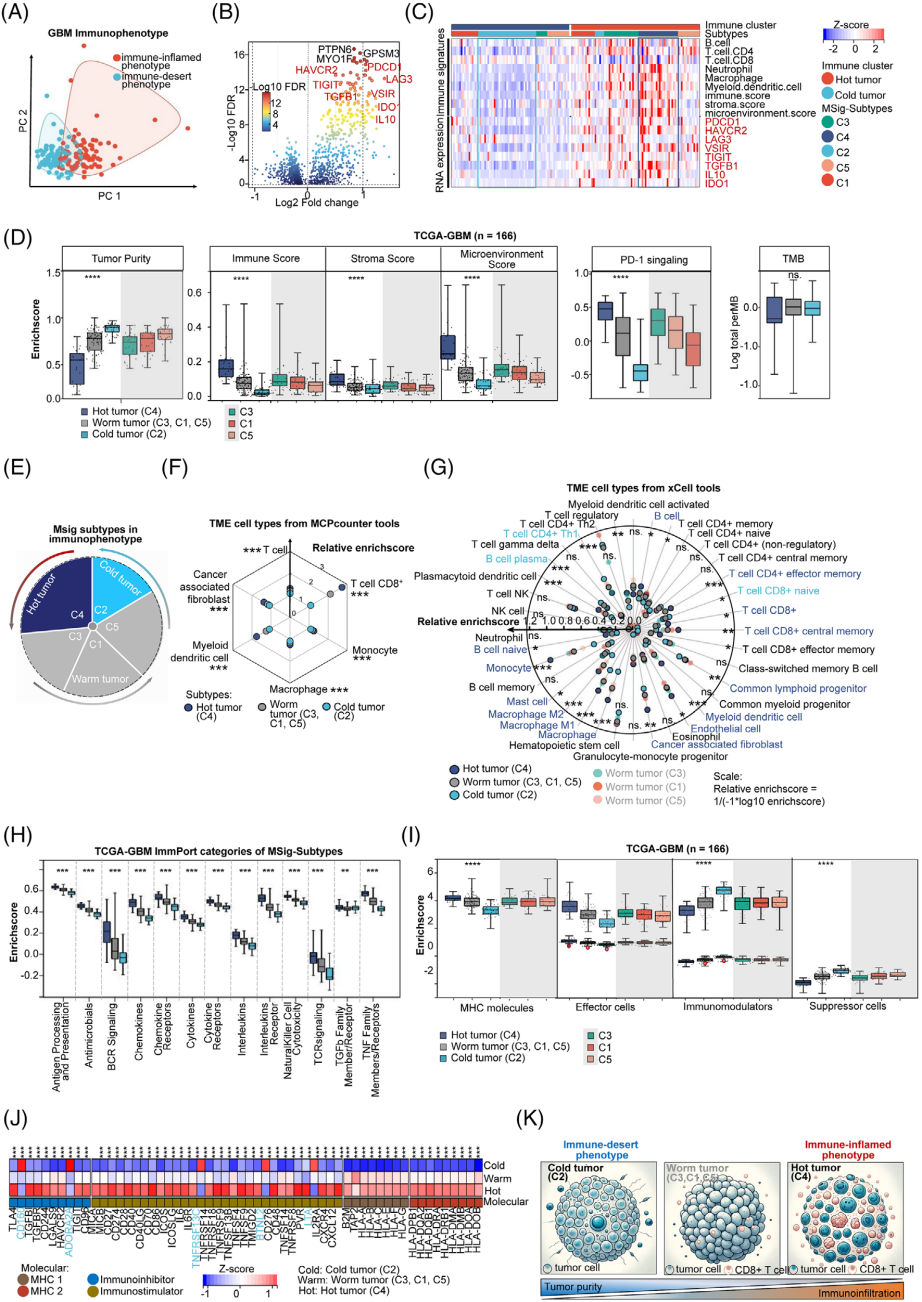
Identification of “cold” and “hot” immunophenotypes of GBM MSig subtypes. (A) PCA plot of tumour‐infiltrating immune cells in GBM based on TIMER analysis, identifying two immunophenotypes via consensus clustering. (B) Analysis of differentially expressed genes (DEGs) between 'immune‐inflamed' and ‘immune‐desert’ phenotypes, highlighting the three most significantly different genes (lowest FDR) in black and immunoinflammatory genes in red. (C) Heatmap showing cell types from TIMER and key immune‐related marker gene expression across the two “immune‐inflamed” phenotypes, “immune‐desert” phenotype, and five MSig subtypes. (D) Comparison of tumour purity, immune scores, stroma scores, microenvironment scores, PD‐1 signalling enrichment, and TMB scores between ‘immune‐inflamed’ vs. ‘immune‐desert’ phenotypes and five MSig subtypes. Significant differences are marked with **** (*p* < .0001); ‘ns’ indicates no significant difference. (E) Classification of patients in MSig subtypes according to immunophenotype, with C4 as hot tumours, C2 as cold tumours, and C3, C1, and C5 as warm tumours. (F, G) Relative enrichment scores of TME cell types from MCPcounter and xCell across ‘immune‐inflamed’ vs. ‘immune‐desert’ phenotypes. (H) ImmPort categories enrichment scores across hot, warm, and cold tumours; significance was assessed using ANOVA.test (***p < .001, **p < .01). (I) Immunophenotype enrichment scores for MHC molecules, effector cells, immunomodulators, and suppressor cells across hot, warm, and cold tumours; evaluated using ANOVA test (****p < .0001). (J) Analysis of DEGs related to MHC I, MHC II, immunoinhibitory, and immunostimulatory molecules between hot vs. cold tumours, analyzed by limma. Blue markings indicate high expression in cold tumours (**p < .001). (K) Schematic representation of differences in GBM immune subtypes and the immune escape mechanism of cold tumours.

To identify potential clinical implications, we analyzed the immune cell landscape of tumour antigen presentation and immunomodulators across five MSig subtypes. First, we classified two immunophenotypes: the immune‐desert and immune‐inflamed phenotypes based on immune cell types from TIMER^79^ using Consensus Clustering^42^ (Figure 7A,B; Table S17). The immune‐inflamed phenotype showed a higher immune score with significant upregulation of B cells, CD4^+^ T cells, neutrophils, macrophages, and myeloid dendritic cells (Figure 7C), along with immunomodulators (PTPN6, GPSM3, and MYO1F) and immunoinflammatory genes (PDCD1, HAVCR2, LAG3, VSIR, TIGIT, TGFB1, IL10, and IDO1) (Figure 7B, C). Second, we identified corresponding MSig immunophenotypes. The immune‐inflamed phenotype included all C4 subtypes, about 50% of C1 and C5 subtypes, 74% of the C3 subtype, and 14% of the C2 subtype (Figure 7C). Consistently, we identified “hot” C4 tumours exhibiting the highest T‐cell infiltration (Figure 7C) and aberrant PD‐L1 signalling (Figure 7D) compared with the “cold” C2 subtype. The C3, C1, and C5 subtypes belonged to “warm” intermediate levels between C2 and C4, as evidenced by tumour mutational burden (TMB) did not show significant differences among C1, C3, and C5 subtypes (Figure 7D,E). These immune classifications were supported by significant differences in tumour purity, immune scores, stroma scores, and overall microenvironment scores among the hot (C4), warm (C3, C1, C5), and cold (C2) tumours (Figure 7D; Figure S6A). Furthermore, we quantify the immune cells across MSig subtypes using *MCPcounter* ^49^ and *xCell*,^45^ showing the significant enrichment of CD8^+^ T cells, monocytes, macrophages, and myeloid dendritic cells in the hot (C4) tumour. In contrast, the cold (C2) tumour showed the enrichment of CD8^+^ naive T cells, CD4^+^ Th1 T cells, and B cell plasma (Figure 7F,G). We analyzed the prognostic significance of immune cells and identified that CD8^+^ naive T cells were positively associated with better survival outcomes (Figure S6B). Conversely, the high enrichment of astrocytes, fibroblasts, and epithelial cells in the C4 subtype is associated with poorer prognosis (Figure S6B–D), highlighting the cell types’ determination for predicting patient outcomes. Indeed, we identified 13 ImmPort categories with significant differential enrichment scores across subtypes using GSVA to ImmPort^51^ categories (Figure 7H; Figure S6E; Table S19). Notably, C4 has the highest enrichment in all these immune‐related processes, with high expression genes within these processes listed in Figure S6F.

Next, to predict immunotherapy response, we analyzed the Immunophenoscore^80^ (IPS) by comparison with major histocompatibility complex (MHC) molecules, immunoinhibitors, and immunostimulators across MSig subtypes. Specifically, the hot tumour (C4) has the highest MHC score, while the cold tumour (C2) displayed the highest suppressor cell (SC) and immunomodulator (CP) enrichment scores (Figure 7I; Figure S6G; Table S20). Notably, the C4 subtype significantly upregulates 8 MHC‐I‐related and 9 MHC‐II‐related antigen‐presenting molecules, 6 immunoinhibitors, and 28 immunostimulators (*p* < .001; Figure 7J). The C2 subtype specifically showed higher expression of immunoinhibitors (CD160, ADORA2A) and immunostimulators (TNFRSF13C, BTNL2, LTA). In sum, C4 subtype upregulates immune processes and abundant immunomodulatory molecules, while C2 cold tumours showed low CD8^+^ T cell enrichment resulting from the downregulation of MHC molecules and the upregulation of immunoinhibitors. Our study may facilitate the prediction of responsiveness to immunotherapy.

### 3.8 MicroRNAs’ landscape of GBM MSig subtypes

**FIGURE 8:**
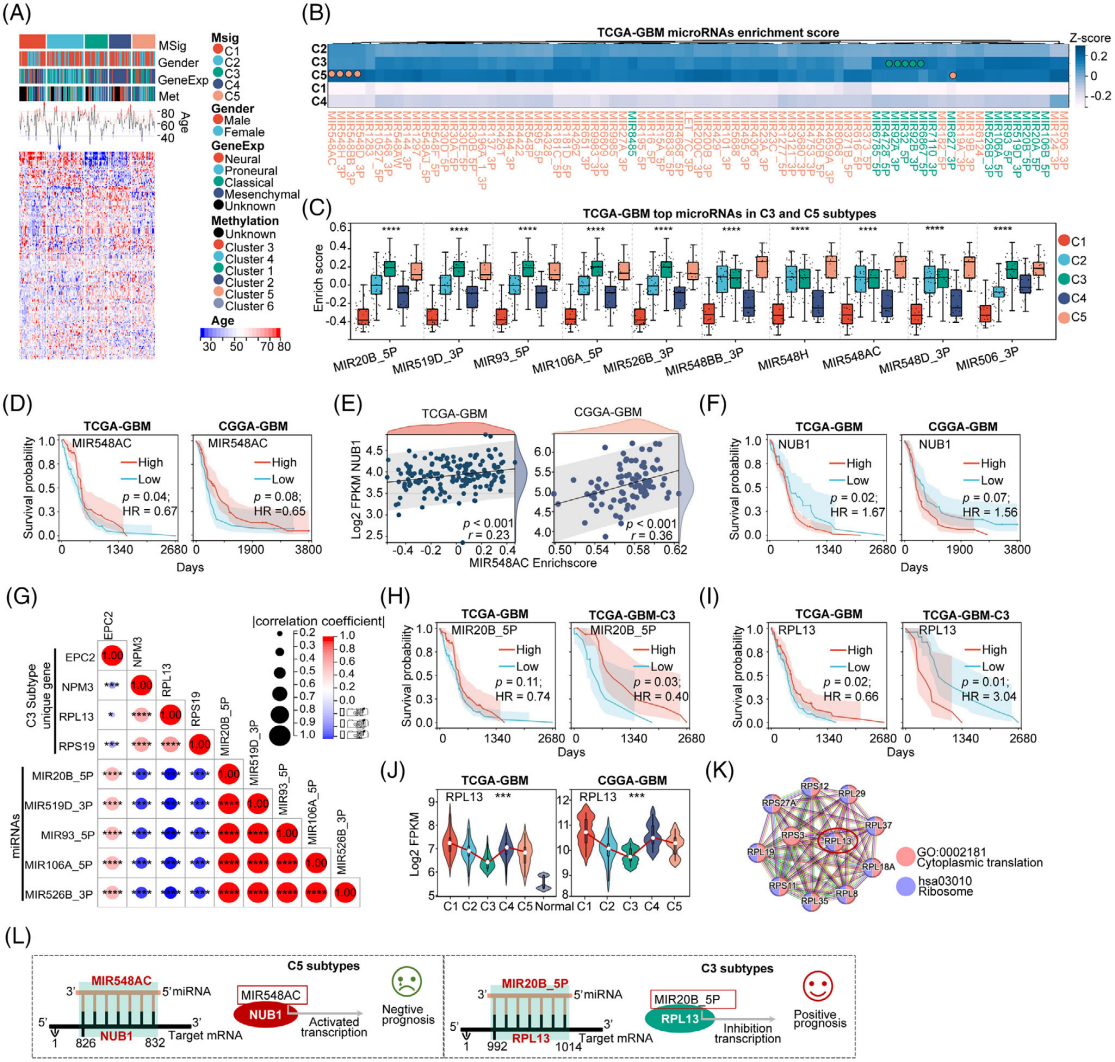
MicroRNA landscape of GBM MSig subtypes. (A) Enrichment scores for GSVA‐predicted microRNAs (*n =* 3703) based on MSig DB across five MSig subtypes. (B) Identification of 79 microRNAs with significant differential abundance across the five MSig subtypes, showing the highest abundance in C3 (green) and C5 (orange) subtypes; ANOVA *p* < .05. (C) The top five most abundant microRNAs in C3 and C5 subtypes across the five MSig subtypes. (D) Kaplan–Meier survival analysis for patients with MIR548AC abundance in TCGA‐GBM and CGGA‐GBM, using the log‐rank test to calculate hazard ratios (HR). Group cutoff was determined by the median of high and low scores. (E) Correlation between MIR548AC abundance and NUB1 gene expression in TCGA‐GBM and CGGA‐GBM datasets. (F) Survival analysis for patients with NUB1 expression in TCGA‐GBM and CGGA‐GBM, using Kaplan–Meier survival analysis, the log‐rank test, and group cutoff by the median of high and low expression levels (HR: hazard ratio). (G) Correlation between the top five most abundant microRNAs and gene expression (NPC2, NPM3, RPL13, and RPS19), with ****p < .0001. (H) Survival analysis for patients with MIR20B_5P abundance in TCGA‐GBM and C3 subtypes, using Kaplan–Meier survival analysis, the log‐rank test, and group cutoff by the median of high and low scores (HR: hazard ratio). (I) Kaplan–Meier survival analysis for patients with RPL13 expression across five MSig subtypes of TCGA‐GBM and C3 subtypes, using the log‐rank test and group cutoff by the median of high and low expression levels (HR: hazard ratio). (J) Analysis of RPL13 expression across five MSig subtypes of TCGA‐GBM and CGGA‐GBM. (K) Protein–protein interactions of RPL13 were analyzed using STRING (https://string‐db.org). (L) MIR548AC and MIR20B_5P were identified as potential therapeutic targets for the C3 subtype.

Next, we analyzed microRNA profiles across five MSig subtypes among 2598 microRNA targets from MSig DB (MIR, regulatory gene sets), and identified 79 microRNA targets with significantly different abundances across the five MSig subtypes (ANOVA, *p* < .05, Figure 8A,B; Table S22). Notably, subtypes C3 and C5 exhibited higher abundances of these 79 microRNAs, whereas C1 and C4 exhibited lower levels (Figure 8B; Figure S7A). We further identified the top five abundantly expressed microRNAs in subtypes C3 and C5. Four of the top microRNAs belong to the MIR548 family in subtype C5 (Figure 8C). Survival analysis revealed that a lower abundance of MIR548AC (representative of the MIR548 family) correlates with poorer prognosis, indicating its potential as a prognostic marker (Figure 8D). We found a significantly positive correlation between MIR548AC and NUB1 expression (Figure 8E), which is consistent with NUB1 as a target gene of MIR548AC (Figure S7B) by Target Scan.^81^ High NUB1 expression correlates with poorer outcomes (Figure 8F), and PPI analysis indicated that NUB1 is involved in proteolysis, deubiquitylation, and the Parkin‐ubiquitin proteasomal system pathway (Figure S7E). Thus, targeting MIR548AC to reduce NUB1 may represent a promising therapy for the C5 subtype.

Additionally, the top five highly expressed microRNAs (Figure 8C) were highly correlated with the expression of NPC2, NPM3, RPL13, and RPS19 (Figure 8G) in the C3 subtype. MiRanda^82^ predicted that MIR20B_5P targets RPL13 (Figure S7C). Low abundance of MIR20B_5P and high expression of RPL13 (involved in cytoplasmic translation and ribosome functions, Figure 8K) are associated with poorer prognosis (Figure 8H,I). Notably, oncogenic RPL13 is found in C3 but not in other subtypes (Figure S7D). Therefore, upregulating MIR20B_5P to reduce RPL13 expression could serve as a targeted therapy for C3 (Figure 8L). In summary, our research highlights the complex regulatory roles of microRNAs across MSig subtypes, which paves a new avenue for developing personalized therapeutic strategies for GBM.

### 3.9 DNA methylation landscape of GBM MSig subtypes

**FIGURE 9:**
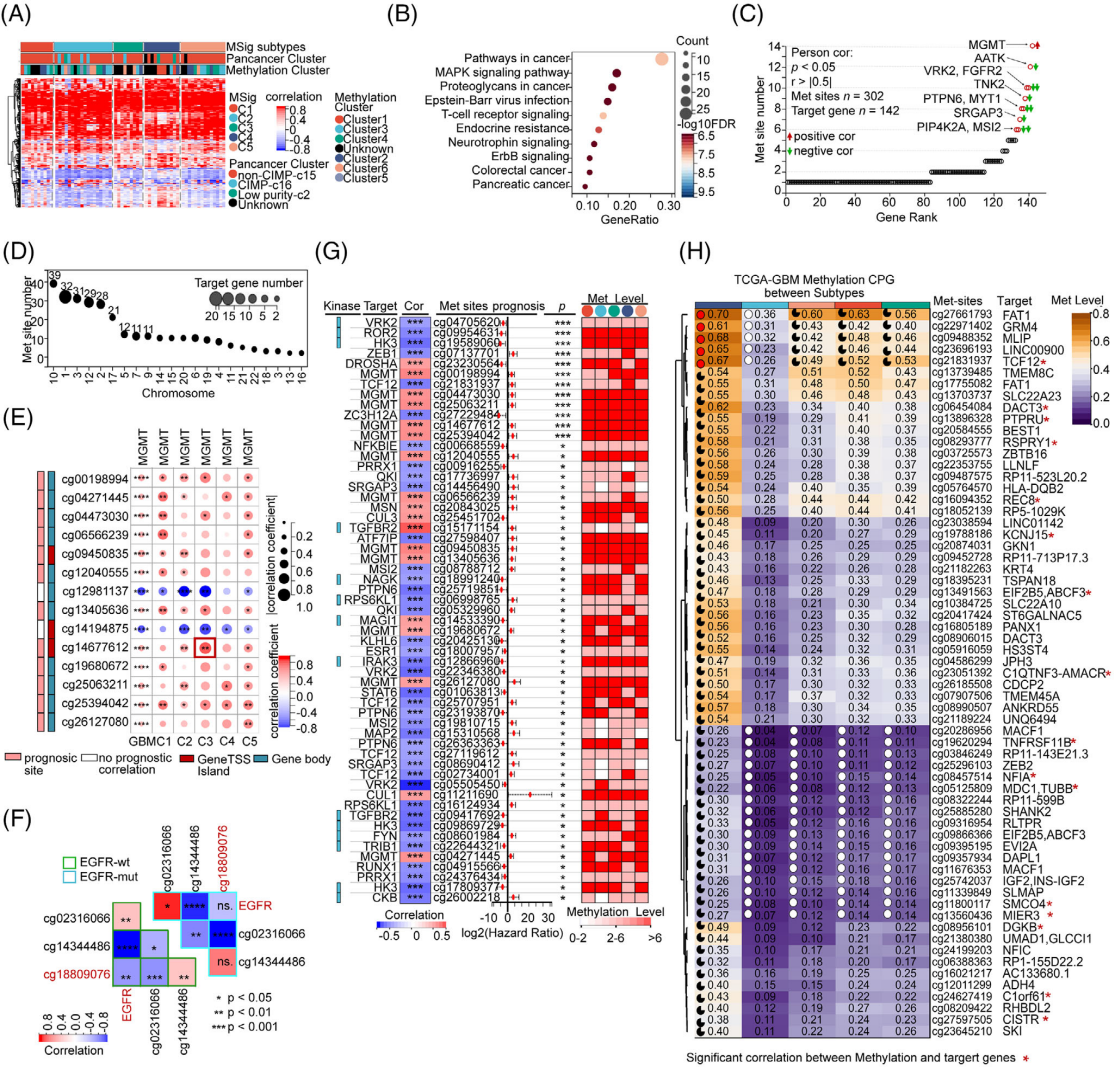
DNA methylation landscape of GBM MSig subtypes. (A) Correlation analysis between methylation sites and genes (methylation‐gene pairs list), with significant thresholds (*p* < .05, |*r*| > .5). Includes counts of genes (142) and methylation sites (302). (B) KEGG pathway enrichment for methylation regulatory genes (methylation‐gene pairs list; *n =* 142). (C) Methylation site count: examines the number of methylation sites linked to target tumour genes. (D) Chromosomal distribution: assesses the distribution bias of methylated sites across chromosomes. (E) Investigation of the relationship between methylation sites and target MGMT expression across all five MSig subtypes. (F) EGFR methylation‐expression pairs: identify three distinct methylation‐expression patterns on EGFR, categorizing patients into EGFR‐wildtype (WT) and EGFR‐mutant (MT). (G) Prognostic methylation sites: analyze 57 prognostic methylation sites across five MSig subtypes. Blue highlights indicate the 14 target genes as kinases. (H) Methylation site abundance: analyzes 63 methylation sites showing significant differences between MSig subtypes (ANOVA, *p* < .05). ‘*’ indicates significant correlations with target genes.

Other potential diagnostic and treatment biomarkers are the status of DNA methylation,^83^, ^84^, ^85^ which adds a methyl group to the cytosine base of DNA, especially at CpG sites.^84^ Hypermethylation silences tumour suppressor genes, promoting uncontrolled cell growth,^86^ while hypomethylation in repetitive DNA regions induces genetic instability and oncogene activation.^87^ First, to analyze the effect of methylation on gene expression, we focus on 63 patients from the DNA methylation dataset of GBM (*n =* 143), matching the RNA sequencing dataset. From 15 620 methylation sites in the tumour‐related genes^88^ (*n =* 1140), we found 302 regulatory methylation sites significantly influencing the expression of 142 tumour‐related genes (*p* < .05, |*r*| > .5) (Figure 9A; Table S23). These 302 regulatory methylation sites significantly impact pathways in cancer, the MAPK signalling pathway, and proteoglycans (Figure 9B). Notably, 42% of the 142 methylation‐regulated tumour genes are controlled by multiple methylation sites, especially MGMT (14 sites), AATK (12 sites), VRK2, and FGFR2 (10 sites) (Figure 9C). We found the clustering of methylation sites on the top 3 chromosomes 10, 1, and 3, indicating chromosomal preference in loci distribution (Figure 9D). Interestingly, 14 methylation sites in the MGMT gene were found (Figure 9E). Notably, the site of cg14677612 in the promoter region of the MGMT gene showed a strong positive prognostic correlation with its expression, indicating the C3 subtype sensitive to TMZ treatment (Figure 9E). We also identified cg02316066 positively (*r* = .53), while cg14344486 and cg18809076 negatively (*r* = −.79 and *r* = −.54, respectively), correlated to EGFR expression (Figure 9F), highlighting potential targets for tumour progression driven by high EGFR expression. Notably, the significant negative correlation between cg18809076 and EGFR expression was observed in patients without EGFR mutations, but not in patients with EGFR mutations (Figure 9F).

Second, we identified 57 prognostic sites that correlate with these 302 regulatory methylation sites, of which 14 sites are in kinase genes (Figure 9G). Correlation analysis between methylation sites and gene expression across the five GBM subtypes showed MSig subtype‐specific regulation preference (Figure S8A). Specifically, the C3 subtype shows a positive correlation between cg10458494 and KNDC1 expression (Figure S8A,B). cg6632603/MTOR and cg12664938/LRRK2 pairs are positively correlated in C5, but not in other subtypes (Figure S8B). High MTOR expression correlated with poorer prognosis, while high LRRK2 correlated with better prognosis (Figure S8C–F). Additionally, we identified 63 prognostic methylation sites with significant expression differences across five MSig subtypes, with the C4 subtype exhibiting hypermethylation levels compared with other subtypes (Figure 9H; Table S24). Notably, the top four hypermethylation sites (cg27661793, cg22971402, cg09488352, cg21831937) were correlated to poorer prognosis (Figure S8G). Specifically, cg21831937 (hypermethylation in C4) negatively correlated with TCF12 gene expression (reduced TCF12 correlated with poorer prognosis) (Figure S8H–J). The PPI analysis shows TCF12 is involved in the regulation of cell differentiation and transcriptional misregulation (Figure S8K), highlighting the potential role of cg21831937 in inhibiting tumour progression by regulating TCF12 expression (Figure S8L).

### 3.10 Cell‐line‐based drug selection for GBM MSig subtypes

**FIGURE 10:**
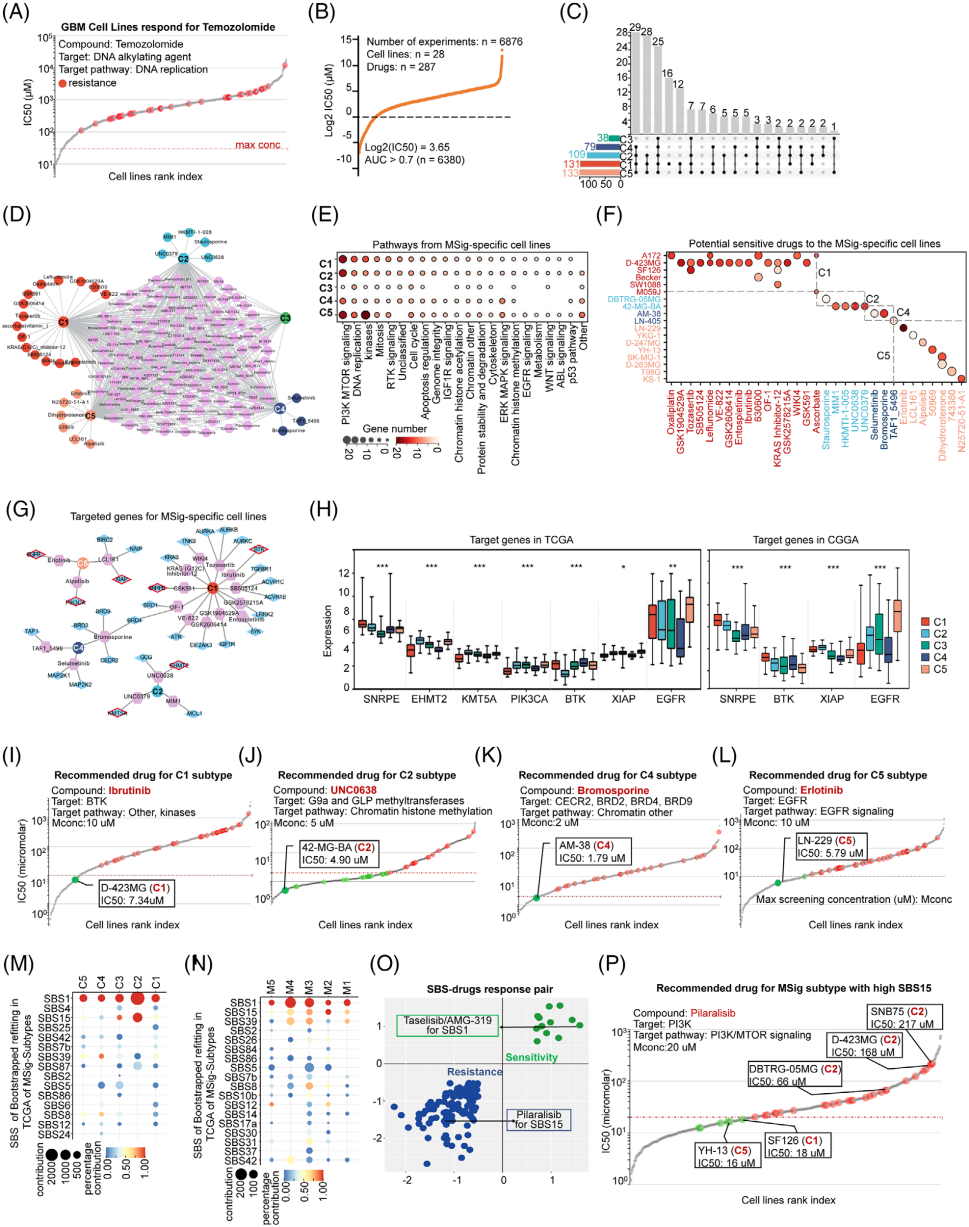
Cell‐line‐based drug selection for GBM MSig subtypes. (A) Analysis of temozolomide response utilizing data from the GDSC database. (B) Drug sensitivity evaluation in GBM cell lines, including 6876 experiments with 287 targeted drugs. (C) Identification of overlapped sensitive drugs from the GDSC database across the five MSig subtypes. (D) Subtype‐specific networks were constructed to identify interactions among sensitive drugs for each MSig subtype. (E) Pathway analysis was conducted for target genes corresponding to sensitive drugs across all five MSig subtypes. Circle size and colour were used to indicate the number of involved genes, enhancing visual understanding. (F) The sensitivity of MSig‐specific drugs to respective cell line subtypes was assessed, represented by circle sizes denoting IC50 values and colours reflecting AUC values. (G) Target genes corresponding to MS subtype‐specific drugs were identified. Those with verified expression in the subtypes were highlighted (marked in red). (H) A compilation of target gene expressions was created, as indicated in Figure 10G, covering all five MSig subtypes within the TCGA and CGGA‐GBM datasets. (I–L) Recommendations for suitable drugs targeting GBM cell lines in GDSC were made for specific C subtypes (C1, C2, C4, and C5). Drugs were categorized as sensitive (green dots) or resistant (red dots), with IC50 values provided. (M, N) Single‐base substitution signatures (SBS) from COSMIC v3.2 were analyzed, focusing on the bootstrapped refitting of patients across five TCGA MSig subtypes and C2 sub‐subtypes. (O) Mapping of SBS–drug response pairs was performed, with sensitivity denoted by green dots and resistance indicated by blue dots corresponding to specific SBS markers. (P) Pilaralisib was recommended for patients exhibiting high SBS15 in GDSC. This recommendation was based on predictions from the BP neural network regarding cell line subtypes.

Next, we try to identify the potential drug selection for MSig subtypes and analyze the sensitivity of cell lines to targeted drugs using the Genomics of Drug Sensitivity in Cancer (GDSC) database.^89^ First, drug sensitivity was assessed using GDSC IC50 values (72 h viability assays). We defined drug effectiveness responses by AUC > .7, and found TMZ‐resistant MSig subtypes for potential clinical implications (Figure 10A). Second, we analyzed 28 GBM cell lines through the MSig subtype predictor and tested their sensitivity to 287 targeted drugs across 6876 experiments in GDSC. We found that 6380 experiments have a higher AUC value (area under the dose‐response curve, >.7) (Figure 10B, Table S25). Subtype‐specific analysis of drug sensitivity revealed: the C1 subtype was sensitive to 131 drugs, C2 to 109, C3 to 38, C4 to 79, and C5 to 133, 25 universal drugs effective across all GBM subtypes, and 31 specific drugs to subtypes (C1: 16, C2: 5, C4: 3, C5: 7), offering treatment options from universal applicability to subtype‐targeted strategies (Figure 10C–D; Table S26). Pathway analysis of genes targeted by sensitivity drugs showed the enrichment of the PI3K‐MTOR signalling pathway in C1, C2, C4, and C5 subtypes, with the C4 subtype showing ERK and MAPK pathways enrichment and C5 dominating kinase targets (Figure 10E; Table S27). We showed 31 sensitivities of MSig‐specific drugs to the corresponding cell line subtypes (Figure 10F). However, drug recommendation of IC50 values from the GDSC in vitro dataset and its capability to penetrate the blood–brain barrier may need further validation.

Next, we showed targeted genes by MSig‐specific drugs, such as Bromosporine, as an MSig‐specific drug to the C4 subtype targeting BRD2, BRD4, BRD9, and CECR2; C5 subtype‐specific drugs target BIRC2, NAIP, and XIAP (Figure 10G). We extracted these target genes’ expression values across the five MSig subtypes of the TCGA‐GBM dataset. SNRPE, EHMT2, KMT5A, PIK3CA, BTK, XIAP, and EGFR have significant expression differences across MSig subtypes (Figure 10H). For example, EGFR, the target gene of C5‐specific drug Erlotinib, indeed has the highest expression in the C5 subtype, verifying our MSig‐specific drug predictions (Figure 10G–H). This comprehensive analysis based on MSig‐specific cells, drug, and target gene expression suggests MSig‐specific targeted first‐line treatment options, such as Ibrutinib (target BTK) for C1, UNC0638 (target EHMT2) for C2, Bromosporine (target BRD2, BRD4, BRD9, and CECR2) for C4, and Eritotinib (target EGFR) for C5 (Figure 10G), validated by sensitivity experiments for MSig‐specific cells in the GDSC (Figure 10I–J).

Moreover, we analyzed mutational signatures as markers for drug sensitivity based on the work from Jurica et al.,^53^ which highlighted associations between mutational signatures and drug activity across cancer cell lines. We analyzed their relative contributions to previously reported SBSs from COSMIC v3.2^40^, ^41^ for the five MSig subtypes by bootstrapping 96 types of trinucleotide substitutions (Figure 10M,N). This revealed distinct mutational signature contributions across the MSig‐subtypes, with SBS1 and SBS15 showing notable stability in subtype C2, especially in M4 patients (Figure 10N); unique contributions from MSig‐SBS8 and MSig‐SBS39 in subtype C4 (Figure 10M). By integrating the list of drugs and related specific SBSs from Jurica et al.,^53^ we identified potential therapeutic agents based on sensitivity or contraindication to specific SBSs across MSig subtypes (Figure 10O; Table S28), such as SBS1 demonstrated sensitivity to Taselisib and AMG‐319, suggesting therapeutic potential for patients in C2, especially subtype M4; SBS15, showing resistance to Pilaralisib, indicates limited efficacy for this drug in C2 subtypes (Figure 10O). Notably, cell lines predicted as C2 subtype with high SBS15 were verified resistant to Pilaralisib in GDSC (Figure 10P). This comprehensive list (Table S28) of SBS–drug response pairs presents the potential for personalized strategies in GBM.

### 3.11 Omics features and treatment recommendations for GBM MSig subtypes

By classifying GBM into five subtypes based on the integrated pathway, hallmark, immunity, and biology process, and performing comprehensive multi‐omics analysis, we offer a rich resource for potential treatment strategies (Table 1). To distinguish MSig subtypes and provide corresponding treatment recommendations, we summarized the characteristics of the five subtypes (Figure S9).

### 3.12 C1: Neural‐like subtype

C1 closely matches the GeneExp‐Neural subtype in the transcriptome (Figure 6A, *r* = .90), and these two subtypes share seven of the top ten mutations (EGFR, PTEN, TTN, MUC16, COL6A3, TP53, and GPR98) (Figure S4A–C). PARK7^68^ is significantly upregulated in C1 compared with other subtypes, also indicating its neural‐like characteristics in C1 (Figure S1I). KEGG analysis reveals spliceosome and ribosome pathway activities in C1, indicating a potential therapeutic pathway (Figure 5B). Drug sensitivity analysis from GDSC on C1 cell lines (A173, M059J) identified Ibrutinib as a promising therapeutic agent for this GBM subtype (Figures 3O and 10I).

### 3.13 C2: Tumour driving subtype

C2 subtype closely matches the GeneExp‐proneural subtype in transcriptome (Figure 6A, *r* = .92), and these two subtypes share six of the top ten mutated genes (Figure S4C). Notably, co‐occurring and mutually exclusive tumour driver mutations (IDH1, TP53, and EGFR) could be used to predict the prognosis of patients (Figure 4C–E). A mutation site at P.A289 of the EGFR gene significantly upregulates EGFR expression in C2 subtypes, providing a new option for EGFR combination therapy (Figure 4H). Pathway analysis reveals the enrichment in cell cycle and Wnt signalling pathways of C2, further confirming the tumour‐driving characteristics (Figure 5B,E). Mutational signature reveals the highest mutation rate closely resembling SBS1(an aging‐associated signature), and based on Taselisib shows sensitivity to patients with SBS1, suggesting it as an effective therapeutic option for treating C2 subtypes (Figure 10M,N). C2, characterized as a cold tumour with reduced immune cells and antigen presentation (Figure 7G), exhibits high expression of immunoinhibitor CD160 (Figure 7K), suggesting targeting CD160 provides a novel immunotherapy strategy for patients of C2.

### 3.14 C3: Premature tumour evolution subtype

C3 subtype has low tumour evolution in the reconstruction of tumour heterogeneity of GBM MSig subtypes in time dimensions (Figure 6B). Pathway enrichment analysis showed C3 is lowest in DNA repair and MYC targets compared with other MSig subtypes (Figure 5E), indicating C3 is relatively stable during tumour growth and proliferation. Notably, the microRNA MIR20B_5P inhibits the expression of PRL13 (Figure 8N), with its high expression only in C3 correlating with poorer prognosis (Figure 8H; Figure S7D), emphasizing the heterogeneity within GBM and a unique treatment target in the C3 subtype. Importantly, the prognostic site cg14677612 in the promoter region of the MGMT gene has the strongest positive correlation with MGMT expression in the C3 subtype, indicating the C3 subtype may be most sensitive to TMZ treatment (Figure 9E).

### 3.15 C4: Hot tumour subtype

The C4 subtype with the worst prognosis among the five subtypes (Figure 2F) is characterized as a hot tumour due to a richer tumour microenvironment and increased IFN‐γ signalling and PD‐L1 expression, indicating a potential for immunotherapy response (Figure 7B). Notably, despite being a hot tumour with an active immune response, the C4 subtype also shows significant upregulation of epithelial–mesenchymal transition (EMT), angiogenesis, and hypoxia (Figure 5E), indicating its higher malignancy and potential for aggressive growth and metastasis. Furthermore, the C4 subtype showed high methylation (Figure 9I), especially the cg21831937 site significantly reduces the expression of the TCF12 gene (Figure 9J–L), offering a potential target to improve patient outcomes. GDSC drug sensitivity experiments recommend Bromosporine as the recommended effective drug for the C4 subtype (Figure 10K), which is based on the subtype molecular profile and its interaction with the tumour microenvironment, emphasizing the importance of personalized medicine in the treatment.

### 3.16 C5: Classical tumour subtype

The C5 subtype, defined as a classical tumour, is closely correlated with the GeneExp‐Classical subtype (Figure 6A). The C5 subtype shows significant activity in classical oncogenic pathways, especially the PI3K/AKT/mTOR, along with the E2M target, G2M checkpoint, and MTORC1 signalling pathways (Figure 5E). The methylation regulatory sites of MTOR in the C5 showed the potential for epigenetic therapy targets (Figure 9G,H). C5 subtype shows significant enrichment of MIR548 family (Figure 8A), which binds to NUB1 and activates transcription, therefore promoting GBM progression (Figure 8N). Erlotinib has been identified as an effective drug for treating the C5 subtype of GBM cell lines based on GDSC (Figure 10L). In sum, the C5 subtype, characterized by classical tumour characteristics, benefits from therapies targeting classical signalling pathways.

## 4 DISCUSSION

The integration of multi‐omics data represents a pivotal advancement in addressing molecular understanding of GBM. This approach facilitates GBM classification, revealing molecular heterogeneity that supports the identification of distinct subtypes with unique prognostic and therapeutic implications.^90^ Moreover, multi‐omics analyses uncover the intricate molecular mechanisms underpinning GBM pathogenesis, including aberrant signalling pathways, genomic alterations, and epigenetic modifications, thus highlighting novel therapeutic targets.^6^, ^90^ This integrated perspective also significantly contributes to the discovery of biomarkers for patient stratification and the prediction of treatment response, laying the groundwork for personalized medicine in GBM.^5^, ^16^ Importantly, multi‐omics studies provide insights into the tumour microenvironment and immune evasion, offering new avenues for immunotherapy.^91^ Additionally, multi‐omics also guides adaptive therapeutic strategies by understanding treatment resistance mechanisms.^92^ Our study is consistent with previous key findings (e.g., EGFR/AC‐like, immune‐hot/MES‐like) in MSig subtypes.^17^ Our framework complements single‐cell studies by linking bulk omics to potential therapies, bridging the gap between heterogeneity and clinical translation. Importantly, the MSig subtypes capture patients’ phenotypes of the transition (e.g., C3's transitional signature or C4's immune–microenvironment interplay). Future integration of single‐cell data may further delineate subtype‐specific hybrid states and profiling to track state transitions during treatment and optimize combination therapies.

Multi‐omics profiling provided a deeper exposition of global GBM and identified new biomarkers with cis‐regulation between CNV, mRNA, and protein. We identified 33 abnormal molecules pivotal for GBM initiation, progression, and treatment responsiveness, underscoring their potential as therapeutic targets. Previous studies of GBM using single omics have pinpointed molecular anomalies such as IDH1, TP53, PTEN, EGFR, and PIK3CA, impacting tumour progression and survival prognosis.^31^, ^32^, ^93^, ^94^, ^95^, ^96^ Our study enriches the current GBM biomarker repertoire by identifying PARK7 and INPP4B based on multilevel CNV, mRNA, and protein analyses. Despite EGFR amplification occurring in 40% of GBM and EGFRvIII mutation in 20%, clinical trials of EGFR tyrosine kinase inhibitors and peptide vaccines (like rindopepimut) in GBM patients have yielded disappointing results.^31^, ^95^, ^97^, ^98^ Our multi‐omics analysis revealed that EGFR overexpression in GBM is interconnectivity regulated by mutations, methylation, and CNV, highlighting the potential of combination therapy targeting the aberrant rewiring signalling pathways. Additionally, Zhang et al.^99^ reported that inhibiting miR‐1193 induces synthetic lethality in GBM cells lacking DNA‐dependent protein kinase catalytic subunit (DNA‐PKcs). Corrales‐Guerrero et al.^100^ found that RRM2 inhibition radiosensitizes GBM, revealing synthetic lethality with CHK1 targeting. We identified a favorable co‐mutation pattern with TP53 and ATRX co‐mutations, which improved patient survival, suggesting their co‐mutagenesis as a new therapeutic strategy. Functional assays would be required to test the reasoning of TP53/ATRX co‐mutation correlated with improved outcomes. Furthermore, mutational signatures reveal the diversity of mutational processes underlying cancer development, with potential implications for understanding cancer aetiology, prevention, and therapy.^53^, ^54^, ^70^, ^101^, ^102^ We delineated total GBM's mutational signature into six novel GBM‐SBSs and revealed potential exogenous factors, such as deamination of 5‐methylcytosine and tobacco smoking, as environmental triggers.^40^, ^41^

MSig subtypes reflect the correlation between GBM heterogeneity and patient prognosis, providing new insights into tumour evolution and immunity. GeneExp‐subtypes revealed that GBMs in specific subtypes arise from diverse causes or cells of origin.^5^ The classification relying on IDH1 mutations can predict patient outcomes but is only present in rare patients.^103^ MSig‐C4 subtypes showed the worst prognosis (with a median survival of 313 days), demonstrating the strength of our classification in prognosis. We found MSig ‐C1, C2, C4, and C5 closely associated with GeneExp‐Neural, Proneural, Mesenchymal, and Classical subtypes, respectively. Importantly, the novel C3 subtype lacked distinct advantages in various tumour hallmarks, highlighting the clarity of MSig subtypes in identifying featureless GBM patients. Additionally, we highlight key characteristics of each subtype, especially in defining tumour subtypes for immune response correlations. The GBM immune microenvironment involves tumour, immune, and stromal cells, fostering an immunosuppressive milieu hindering anti‐tumour responses. Infiltrating myeloid‐derived suppressor cells, regulatory T cells, and M2‐polarized macrophages facilitate tumour growth and immune evasion through cytokine secretion.^104^, ^105^ GBM immunological features, including PD‐L1 expression and T cell exhaustion, correlate with poor prognosis and immune evasion.^106^ Tumour heterogeneity affects immune recognition, impacting immunotherapy efficacy, emphasizing the need for personalized strategies to overcome GBM's immunosuppressive response and enhance treatment outcomes.^106^, ^107^ MSig ‐C4 subtype exhibits a rich tumour microenvironment with increased IFN‐γ signalling and PD‐L1 expression, whereas C2, as a cold tumour, shows the lowest levels, highlighting the importance of subtype‐specific immunotherapy. Zhang et al.^108^ reported that immune checkpoint blockade therapies have achieved clinical benefit, but many immunecold solid tumours remain unresponsive. Combining immunotherapies to convert cold tumours into hot tumours may offer superior clinical benefits compared with monotherapy in cancer patients. Immunotherapies for C2 require addressing reduced antigen presentation due to MHC molecule downregulation and increased immunoinhibitors; regulating these immune‐related biomarkers may enhance cold tumour responsiveness to immunotherapy. Significantly, our BP neural network model based on ten representative genes effectively predicts CGGA patient subtypes in external datasets and verifies differences in immunity, showing potential for clinical application. Predicting subtypes for cell lines is crucial for forecasting drug sensitivity across MSig subtypes to guide treatment. We offer a list of subtype‐specific drugs that avoid extensive experimental screening in the clinic. We therefore extended validation using the CPTAC GBM proteomic cohort, which confirmed that MSig subtypes preserve their hallmark pathway profiles at the proteome level. Importantly, CPTAC phosphoproteomics provides a future opportunity to dissect phosphorylation‐driven heterogeneity, particularly within the immune‐inflamed/mesenchymal‐like C4 subtype, where distinct signalling modules (e.g., NF‐κB, STAT3) may define clinically relevant subclusters (Figure S5C). Such proteogenomic refinement will further strengthen the clinical translation of the MSig framework. MSig classification also highlights the prognostic significance of TP53, EGFR, and IDH1 mutations in the tumour‐driving C2 subtype and the therapeutic potential of targeting the classical tumour pathway in the C5 subtype. Our MSigDB‐based classification identifies conserved pathway modules that are pharmacologically targetable. For example, C4's immune signature aligns with recent successes in ICI‐treated GBM subsets, and C2's EGFR/kinase axis mirrors clinical responses to tyrosine kinase inhibitors. Future work integrating single‐cell data could further refine these associations. While our MSig subtypes provide a snapshot of GBM heterogeneity, we recognize that cellular plasticity may lead to therapeutic resistance. For instance, EGFR inhibition in C2 could enrich for MES‐like (C4) clones, as observed in single‐cell studies.^17^ Future work should integrate longitudinal single‐cell profiling to track state transitions during treatment and optimize combination therapies.

MSig subtypes delineate tumour evolutionary potential and progressive trends, influencing intratumour heterogeneity and promoting GBM growth, resistance, and invasiveness. Previous studies have underscored the evolutionary aspects of tumour development,^109^, ^110^, ^111^, ^112^, ^113^, ^114^, ^115^ including subclonal genetic and epigenetic changes,^17^, ^116^, ^117^ metastatic dissemination,^118^, ^119^ and the development of therapeutic resistance.^120^, ^121^, ^122^, ^123^, ^124^ Wang et al.^125^ revealed that 63% of patients undergo expression‐based subtype transfer in a highly branched evolutionary pattern. Wang et al.^126^ discovered that recurrent GBMs shift towards a mesenchymal phenotype mediated by activator protein 1. Kim et al.^36^ performed multi‐omic analyses on patient‐derived xenograft models, revealing that inhibiting B‐raf proto‐oncogene kinase reduces the neuronal migration ability in recurrent tumour cells. The relationship between GBM subtypes and evolution also remains unclear. Our study utilized pseudo‐time analysis to map the population evolution pattern of GBM MSig subtypes. We found that C3 represents the initial stage of GBM evolution, resembling normal tissues and exhibiting low levels of DNA repair, oxidative phosphorylation, and MYC targets, indicating tumour immaturity and potential transformation to C2 and C4 subtypes. Importantly, tumour‐driving C2 subtype and immune‐inflamed C4 subtype exhibit significant differences in tumour hallmarks and immune infiltration, underscoring their roles in the evolutionary endpoint of GBM trajectory.

While our mutational signature analysis identified patterns resembling tobacco‐associated SBS4, and TP53‐ATRX co‐mutations showed survival benefit, these remain putative associations. Definitive conclusions require (1) epidemiological confirmation of exposure histories and (2) experimental validation of genetic interactions using perturbation models. While our cell line‐drug associations provide mechanistic insights, clinical translation requires addressing the blood‐brain barrier and immunosuppressive microenvironment. Future studies should (1) employ BBB‐penetrance scoring systems, (2) test combinations with immunomodulators in C4 PDXs, and (3) validate targets like BRD4 in mesenchymal C4 using inhibitors with known CNS activity.

Our study advances beyond prior GBM classification frameworks^5,^ ^17^ in both methodology and clinical implications. First, unlike single‐omics or gene expression–only approaches, our MSig classification integrates DNA mutations, copy number, DNA methylation, transcriptome, and proteomic data into pathway‐level features. This multi‐omics perspective enables us to capture cis‐regulatory mechanisms (e.g., EGFR, PARK7) that directly connect genomic alterations to protein‐level activity and patient outcomes, thereby providing a mechanistic basis that was underrepresented in previous classifications. Second, we identify a novel transitional subtype (MSig‐C3), which exhibits globally low pathway activity and lies evolutionarily between normal‐like tissue and advanced GBM subtypes. Unlike endpoint‐focused categories, this intermediate state highlights a unique therapeutic opportunity: C3 tumours display high MGMT promoter methylation and are predicted to be temozolomide‐sensitive, suggesting a window for early chemotherapeutic intervention to prevent progression towards aggressive tumour‐driving (C2) or immune‐inflamed (C4) endpoints. Third, we explicitly couple immune phenotypes with tumour evolutionary trajectories, revealing that C4 tumours represent immune‐inflamed “hot” states (PD‐L1/IFNγ upregulated, MES‐like), whereas C2 tumours represent immune‐desert “cold” states (low MHC, CD160 upregulated, PN‐like proliferative). This immune–evolutionary integration provides a rationale for subtype‐specific immunotherapy strategies, such as immune checkpoint inhibitors in C4 or immunomodulatory combinations in C2, which were not apparent from gene‐expression classifications alone. Finally, by systematically integrating drug sensitivity data (GDSC/PRISM) and SBS–drug associations, we move beyond descriptive taxonomy towards clinically actionable recommendations. For example, bromosporine in C4 (BRD4‐targeted, consistent with immune‐inflamed biology), EGFR/PI3K–axis inhibitors such as erlotinib in C5, and Taselisib in C2 patients carrying SBS1 signatures. These findings demonstrate that pathway‐based subtyping not only refines biological stratification but also enables precision‐guided therapy selection. In sum, the MSig framework provides three key advances: (1) multi‐omics pathway integration that mechanistically links DNA → RNA → protein → phenotype; (2) recognition of a transitional C3 state with therapeutic relevance; and (3) explicit connection of immune phenotypes and drug sensitivity to subtype biology. Together, these innovations highlight both the biological novelty and clinical utility of our classification system.

In sum, tumour heterogeneity remains a significant challenge, and emerging methods for single‐cell approaches to heterogeneity provide critical insights into metastatic biology. This study integrates the multi‐omics advances to understand the prognostically classification and treatment of GBM.

We extended the CPTAC proteomic and phosphoproteomic cohort to confirm subtype‐specific pathway classification at the depth of protein level. Future work may need to integrate additional CPTAC cohorts and expanded phosphoproteomic datasets. Second, our trajectory analysis was derived from bulk RNA‐seq, which inherently represents a mixture of tumour, immune, and stromal factors. The averaging effect can obscure rare subpopulations and mask opposing programs, limiting resolution compared with single‐cell lineage tracing. To mitigate this, we focused on pathway‐level enrichment and performed immune deconvolution, which may capture dominant biological programs more robustly than single‐gene signals. Thus, these trajectories can be interpreted as patient‐level evolutionary trends rather than precise cellular lineages. Future studies integrating single‐cell and spatial transcriptomics will be utilized to dissect subtype‐specific transitions (e.g., PN‐like to MES‐like shifts).

Our RPPA analysis was confirmatory only, reflecting the platform's targeted coverage and limited phosphosite resolution. Looking ahead, CPTAC‐level deep proteomics and phosphoproteomics could provide higher resolution of signalling states—e.g., RTK/MAPK, PI3K–AKT–mTOR, NF‐κB/TGF‐β/EMT axes—and thereby refine or subdivide the C4 “hot/mesenchymal” subtype. For instance, phosphosite‐resolved signalling may distinguish immune‐inflamed versus stromal/mesenchymal‐dominant states, or separate tumours with STAT3/TGF‐β–driven EMT from those dominated by ECM remodelling and macrophage programs. Such data could clarify therapeutic vulnerabilities and improve the biological interpretability of C4 while keeping transcriptomics‐based subtype assignment intact.

## 5 CONCLUSION

In this study, we present comprehensive multi‐omics profiles of glioblastoma (GBM), identifying 33 aberrant molecules involved in kinase activity and immune response. We delineated tumour evolution and oncogenic hallmark‐mediated molecular classifications based on integrated pathways, hallmarks, immunity, and biological processes. Subsequent in‐depth analyses of driver mutations, tumour hallmarks, immunophenotypes, population evolution trajectories, and methylation regulation across MSig subtypes provide new perspectives for individualized therapy.

## AUTHOR CONTRIBUTIONS

All authors reviewed and approved the final manuscript. Pei Zhang, Dan Liu, and Lei Dong contributed to the conception, design, and drafting of the paper. Tonghui Yu performed statistical analysis and interpretation of results. Qin Xia, Yanlin Zhang, Lu Zhong, and Xiao Ouyang revised the paper.

## CONFLICT OF INTEREST STATEMENT

The authors declare no conflict of interest.

## CONSENT FOR PUBLICATION

All the authors agreed to publish this paper.

## ETHICS STATEMENT

This study was based on TCGA and CGGA databases, and no animal experiments were conducted.

## Supporting information



Supporting Information

Supporting Information

## Data Availability

All data generated or analyzed during this study are included in this published article and the Supporting Information Tables.
